# Signaling pathways in intestinal homeostasis and colorectal cancer: KRAS at centre stage

**DOI:** 10.1186/s12964-021-00712-3

**Published:** 2021-03-10

**Authors:** Camille Ternet, Christina Kiel

**Affiliations:** School of Medicine, Systems Biology Ireland, and UCD Charles Institute of Dermatology, University College Dublin, Belfield, Dublin 4, Ireland

**Keywords:** Hypoxia, Inflammation, Intestinal stem cells, MAPK pathway, Metabolic reprogramming, Microenvironment, Reactive oxygen species, Small GTPases, KRAS, Colorectal cancer

## Abstract

The intestinal epithelium acts as a physical barrier that separates the intestinal microbiota from the host and is critical for preserving intestinal homeostasis. The barrier is formed by tightly linked intestinal epithelial cells (IECs) (i.e. enterocytes, goblet cells, neuroendocrine cells, tuft cells, Paneth cells, and M cells), which constantly self-renew and shed. IECs also communicate with microbiota, coordinate innate and adaptive effector cell functions. In this review, we summarize the signaling pathways contributing to intestinal cell fates and homeostasis functions. We focus especially on intestinal stem cell proliferation, cell junction formation, remodelling, hypoxia, the impact of intestinal microbiota, the immune system, inflammation, and metabolism. Recognizing the critical role of KRAS mutants in colorectal cancer, we highlight the connections of KRAS signaling pathways in coordinating these functions. Furthermore, we review the impact of KRAS colorectal cancer mutants on pathway rewiring associated with disruption and dysfunction of the normal intestinal homeostasis. Given that KRAS is still considered undruggable and the development of treatments that directly target KRAS are unlikely, we discuss the suitability of targeting pathways downstream of KRAS as well as alterations of cell extrinsic/microenvironmental factors as possible targets for modulating signaling pathways in colorectal cancer.

**Video Abstract**

**Supplementary Information:**

The online version contains supplementary material available at 10.1186/s12964-021-00712-3.

## Background

### The intestinal epithelium

**Fig. 1:**
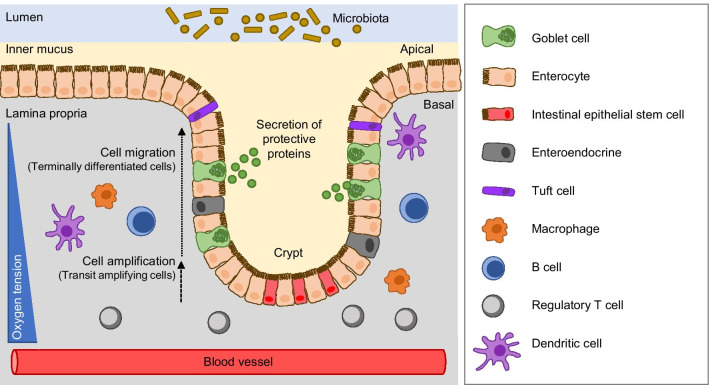
Components of the large intestine/colon. The large intestine is composed of intestinal epithelial cells, which arise from the intestinal stem cells localised at the bottom of the crypts and differentiate into several cell types such as goblet cells, enteroendocrine cells, tuft cells or enterocytes. All of them are playing a role in the homeostasis state of the single layer of the intestinal epithelium to maintain the barrier function. This barrier separates the external (e.g. microbiota) from the internal environment (immune system) which together contribute to the preservation of the intestinal barrier

The large intestine, also known as colon or large bowel, is one of the fundamental parts of the gastrointestinal digestive tract. It acts as a filter, facilitating the uptake of food-derived nutrients, water, electrolytes, and vitamins from the intestinal lumen. The inside surface of the colon or mucosa is made of columnar epithelial cells and unlike in the small intestine, intestinal villi are absent [1]. The intestinal epithelial cells (IECs) shape multitubular invaginations that form crypts, which increase the absorption surface of the tissue. At the base of the crypts, the intestinal stem cell (ISC) niche enables the constant regeneration of the intestinal lining (e.g. enterocytes, endocrines, or goblet cells). These cells can proliferate, differentiate and move upwards, where they are replaced every five to seven days in the human colon [2] (Fig. 1).

The IECs form a continuous epithelium of cells that are tightly linked by different types of cell–cell junctions that assist in maintaining the integrity of the barrier [3]. In this way, the IECs have a dual role in controlling dynamic interactions occurring between the two distinct environments they physically separate. On the one hand, IECs regulate the absorption of water, nutrients, and electrolytes from the external environment (i.e. contents of the lumen). On the other hand, IECs act as a barrier to protect the host tissue from commensal bacteria (microbiota) by preventing the entry of harmful agents or substances (e.g. toxins, microorganisms, etc.). Therefore, IECs are important mediators of homeostasis to enable the establishment of an immunological environment permissive to colonization by commensal bacteria [1]. Notably, IECs are also subject to low oxygen levels due to the proximity of the oxygen-depleted gut lumen, thus experiencing relative hypoxia, or “epithelial hypoxia”, even in the “physiologically” healthy state [4]. This particular environment is essential to the intestinal epithelial barrier function and immune cells activity. Intestinal homeostasis depends on a tightly regulated crosstalk among commensal bacteria, mucosal barrier, immune cells and IECs [5]. However, this homeostasis state can be impaired during inflammation, caused e.g. by bacteria or food, which can promote the development of diseases such as inflammatory bowel disease (IBD) or colorectal cancer (CRC).

### The Ras superfamily and KRAS in colon homeostasis

**Fig. 2:**
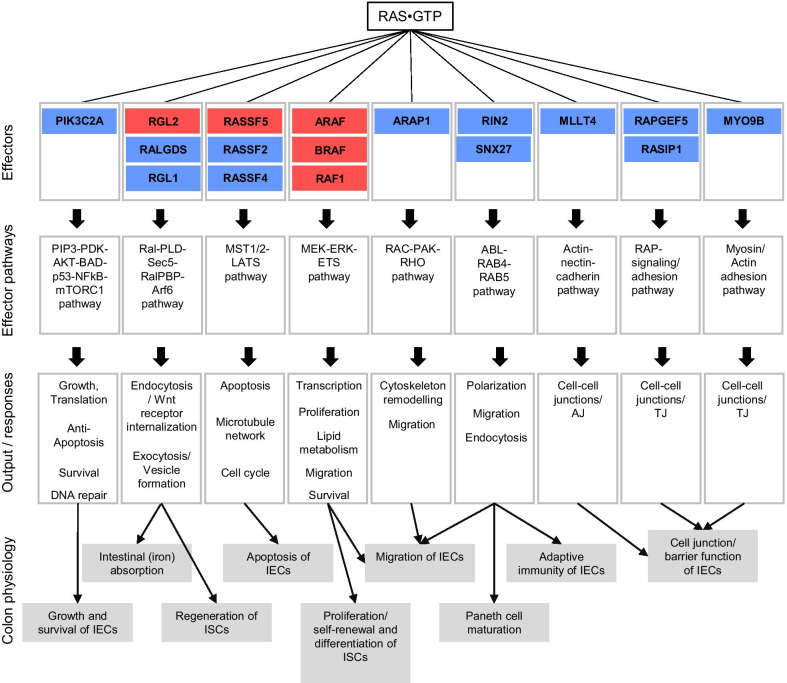
RAS-effector signaling pathways relevant in colon context. Effector proteins are grouped according to functional classes (see [13]). Effectors coloured in red indicate that a significant amount of effector (> 5%) is found in complex with RAS·GTP in normal colon context (based on computational models by Catozzi et al., in press). Effectors in blue indicate that context-dependent membrane recruitment of additional effectors present in effector pathways are needed in order to be significantly in complex with RAS GTP (see Catozzi et al., in press). Effectors are named following their official gene symbol

The RAS superfamily of small GTPases [6] (with RAS, Rho/Rac, Arf and Rab subfamilies) are critical regulators of intestinal epithelial homeostasis and barrier function [7]. At the molecular level, RAS proteins cycle between an inactive state, where they are bound to guanosine diphosphate (GDP), and an active state, bound to guanosine triphosphate (GTP). The transition between the two states is regulated by two main protein families, GTPase-activating proteins (GAPs), which catalyse the hydrolysis of GTP and guanine nucleotide exchange factors (GEFs), which catalyse the exchange of GDP with GTP [8]. The RAS oncoprotein members of the Ras subfamily (HRAS, NRAS and KRAS with its two isoforms, 4A and 4B) are membrane-associated proteins that play a fundamental role in cell signaling. RAS loaded with GTP can interact with multiple effectors such as RalGDS, phosphoinositide 3-kinase (PI3K) or Raf kinase, which are able to control cellular processes such as polarization, adhesion and proliferation [9, 10] (Fig. 2).

KRAS proteins are the predominant isoforms expressed in the colon, in a proportion of ~ 88% (against ~ 4% and ~ 8% for HRAS and NRAS, respectively) [11, 12]. Previously, we generated a quantitative computational model that linked KRAS to downstream effector pathways in colon context [13]. We predicted that Raf effectors are the main effector family in complex with  RAS [13], which induce proliferation/self-renewal and differentiation of ISCs [14]. Further, the effectors RalGDS/RalGDS-like, the RASSF family, afadin (AFDN), and the RIN family of effectors were predicted to form significant amounts of complexes with RAS in physiological normal intestinal epithelium [13]. RalGDS and the activation of Ral GTPases have been shown to be critical for the regeneration of intestinal stem cells [15]. The RASSF-MST-LATS pathway coordinates intestinal regeneration by means of cell proliferation, apoptosis and differentiation functions [16]. AFDN is involved in formation of cell–cell junctions, and thereby controls adhesion between different IECs [17].

### Molecular carcinogenic pathways and subtypes in CRC

CRC is a very heterogeneous disease with different genetic signatures and molecular carcinogenesis pathways linked to various subtypes [18]. CRC arises though multiple genetic events and, historically, at least three molecular pathways are involved in the development and progression of CRC, which are the (1) Chromosomal Instability; (2) Microsatellite Instability; and (3) CpG Island Methylator phenotypes [19]. This classification is mainly based on the molecular drivers (e.g. mutations and amplifications) of the different tumor types, such as KRAS mutants, BRAF V600E, and HER2 amplifications [20, 21]. The heterogeneity of the disease significantly affects the response to targeted treatments of CRC. For example, tumors with mutations in KRAS, NRAS, BRAF, or right-sided tumors do not show any anti-EGFR treatment benefit [18]. Indeed, right side tumors, which more frequently contain KRAS and BRAF mutants compared to left-sides tumors, denote an overall worse prognosis for patients [22].

In 2015, the Colorectal Cancer Subtyping Consortium used a combination of multi-omics (e.g. transcriptomics) and clinical data to define four consensus molecular subtypes (CMSs), which is considered the most robust CRC classification to date [23]. These are: (1) CMS1 (associated with microsatellite instability and upregulation of immune genes, 14%); (2) CMS2 (defined by the canonical ‘adenoma-carcinoma sequence’ with mutations in adenomatous polyposis coli (APC), p53, and RAS, 37%); (3) CMS3 (associated with metabolic dysregulation, i.e. increased glutaminolysis and lipidogenesis, 13%); and (4) CMS4 (associated with epithelial–mesenchymal transition, 23%) [23]. CMS3 is particularly relevant for this review as it shows a clear overrepresentation of KRAS mutations (80%) [24].

### KRAS in colorectal cancer and context-dependent signaling

Among the three human RAS oncogenes (KRAS, NRAS and HRAS), KRAS is the most frequently mutated isoforms in pancreas, lung, and colorectal cancer. Approximately 45% of CRC harbour KRAS mutations, most commonly in the hotspot codons 12, 13 or 61 [25]. Oncogenic mutations render KRAS insensitive to GAP-mediated GTP hydrolysis and lock KRAS in an active GTP-bound conformation; hence this causes disruptive activation of downstream pathways by recruiting specific effectors mediated by KRAS mutant proteins [26]. According to the COSMIC database, KRAS mutations are dominated by G to A transitions at the second base of codons 12 and 13 which give the G12D and G13D mutations (43%) respectively, the G to T transversions at the second base of codon which give the G12V and also the G12C mutations when it occurs at the first base of the codon. The nucleotide to which G is mutated (e.g. G to A) differentially impacts the activity of the protein and the prognosis as well as responses to epidermal growth factor receptor (EGFR)-mediated therapy in CRC patients [27, 28]. In addition, it has been shown that there are isoforms-specific patterns of codon mutations even within the same tissue [29]. In fact, some studies suggest that the position and type of amino-acid exchange influence the transforming capacity of KRAS mutant proteins [27, 30].

**Fig. 3:**
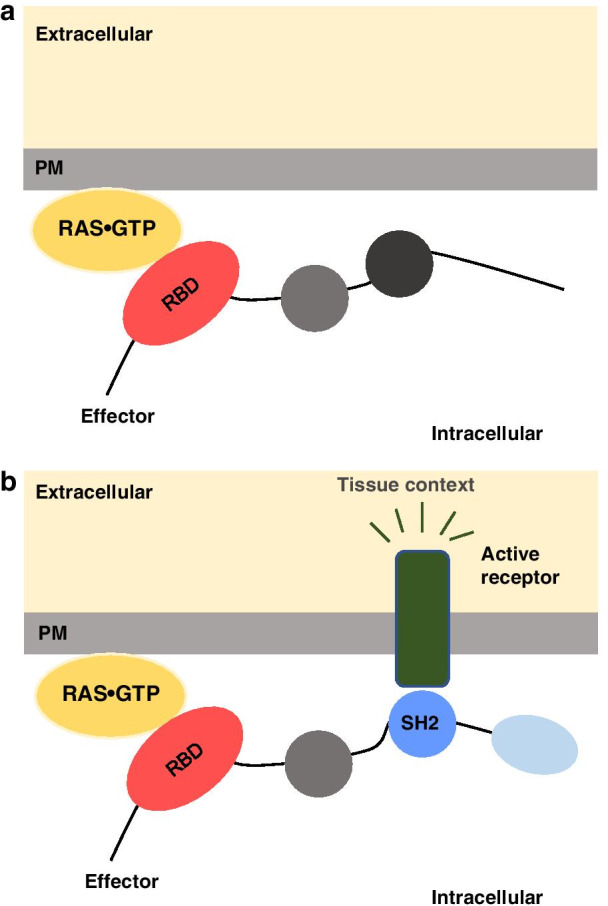
Comparison of Ras effectors binding to RAS•GTP via its Ras binding domain (RBD) or via its RBD and additional membrane-associated domains. **a** Schematic representation of plasma membrane (PM), domain of RAS•GTP (in yellow) and example of an effector with three domains. The RBD of the effector that is used to bind Ras GTP is coloured in red. **b** Similar schematic representation as in (a), where the effector uses an additional domain (coloured in blue) for membrane association. In this example, the domain in blue represents an Src homology 2 (SH2) domain that can bind to a phosphorylated membrane receptor

As shown earlier, changes in the relative abundances of the RAS binding effectors can cause network rewiring and alterations in downstream signaling responses [13]. Furthermore, for some effectors, the formation of Ras-effector complexes at the plasma membrane (PM) is predicted to greatly increase in response to specific conditions (i.e. inputs/stimuli/growth factors) by recruitment to the PM through other domains [31] (Fig. 3). Therefore, rewiring in cancer is proposed to be not only the outcome of a constitutive activation of the Raf- mitogen-activated protein kinase (MAPK) pathway, but the result of all competing interactions that are modulated by signals (e.g. microenvironment) of the tumor. Understanding the cell fates triggered as a result of both, the (micro)-environment/conditions and the type of KRAS mutations, is crucial to better characterize diseases of the intestinal tract, such as inflammatory bowel disease and CRC. Indeed. IECs require a high level of coordination to undergo a series of cell fate decisions during their lifetime, some of which are coordinated by the small GTPase KRAS. In this review, we provide a comprehensive overview on how the environment, stimuli, and growth factors are involved in the intestine physiology and pathophysiology. We highlight—whenever is known—about their link to KRAS-mediated signaling pathways.

## Maintenance of the IECs and role of KRAS signaling in physiological conditions

### Intestinal stem cells and maintenance of intestinal homeostasis

**Fig. 4:**
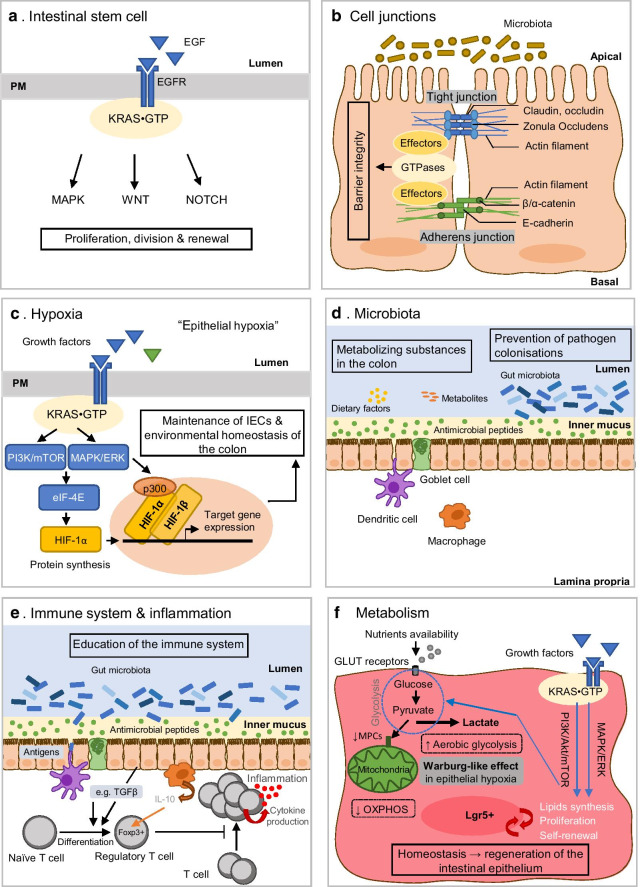
Illustration of intestinal homeostasis and implication of KRAS. **a** Intestinal stem cells (ISCs) are maintaining intestinal homeostasis through different signaling pathways including the RAS/MAPK. **b** Tight junctions and adherens junctions allows intestinal epithelial cells (IECs) to maintain a functional barrier integrity. **c** Epithelial hypoxia maintains IECs homeostasis (barrier function) as well as the environmental colon environment (microbiota and immune cells). **d** The commensal bacteria that colonize the colon are in symbiosis with the host to sustain intestinal balance. **e** Immune cells present in the colon contribute to the prevention of intestinal inflammation by maintaining intestinal homeostasis. **f** Signaling pathways that regulate proliferative cells metabolism (e.g. in ISCs) are essential to maintain self-renewal and proliferation rate of these ISCs, which maintains the integrity of the intestinal barrier

Every five to seven days, the intestinal epithelium is regenerated by cell division and proliferation of ISCs, which differentiate into all intestinal lineages, such as Goblet cells, Paneth cells, endocrines and enterocytes. This rapid turnover maintains the epithelial barrier and homeostasis of the epithelium. ISCs were identified by Barker and colleagues in the small intestine and colon by the marker gene leucine rich G-proteins-coupled receptor 5 (Lgr5 +) [32]. Two types of stem cells have been reported at the crypt level: the homeostatic stem cells that generate new progenitors to renew the epithelium, and the likely quiescent stem cells, which provide a reserve of stem cells in case of injury [33, 34]. The rapidly proliferating stem cell population, that expresses Lgr5 + , is regulated by several pathways such as the wingless-related integration site (Wnt) and Notch pathways. A major role is also played by the MAPK pathway that is activated in response to EGF [35] (Fig. 4a). Indeed, EGF, which is produced by Paneth cells and located in the stem cell niches (where EGF receptors are highly expressed), and others such as signaling molecules found in the environment can control the activity of ISCs [36]. Furthermore, it is well known that mutations in proteins of the MAPK pathway, such as BRAF or KRAS, are involved in the initiation and progression phases of colorectal cancer [37, 38].

ISCs are proposed to be the cells-of-origin for CRC [39, 40], although this ‘stem cell hypothesis’ is still controversially discussed (see [41] for an up-to-date review on this topic). KRAS mutant proteins have a major role in stem cell activities. For example, Le Rolle and colleagues demonstrated that oncogenic KRAS can induce an embryonic “stem cell-like program” to enhance CRC progression from adenoma to carcinoma [42]. Another study showed that oncogenic KRAS can activate cancer stem cell properties in APC-mutated cells (loss-of-function mutations that occur during initiation stages of CRCs) [43]. In conclusion, KRAS is a key player involved in the maintenance of intestinal homeostasis, on one hand, and in driving CRC progression, on the other hand. Hence, through the activation of the MAPK pathway in homeostasis state and the presence of specific mutations in cancer, KRAS seems to be at the interplay between the controlled and the uncontrolled regulation of proliferation in stem cells, possibly leading to cancer.

### Role of cell–cell junctions for intestinal homeostasis

The intestine epithelium is formed by a continuous monolayer of IECs with columnar shape that maximizes surface area for absorption [44]. IECs are tightly linked to maintain a functional and robust barrier integrity between the internal environment (immune system/tissue) and the external environment (microbiota/lumen) [17]. The linkage is mediated by three types of cell–cell junctions: tight junctions (TJs), adhesion junctions (AJs) and desmosomes. These junctions are involved in the maintenance of homeostasis by regulating multiple processes such as the diffusion of ions and solutes, the confinement of bacteria to the lumen, the establishment of cell polarity, and the regulation of intestinal cell proliferation and migration (Fig. 4b). Cell–cell junctions act as signaling hubs at the PM and are part of an interconnected protein–protein interaction network of adhesion complexes [45]. These complexes are enriched in signaling molecules such as small GTPases, their regulators and effectors, and thereby act as the starting point from which multiple intracellular signaling pathways are triggered [46]. Members of the RAS subfamily are known to regulate cell proliferation, polarization and survival [47], however, compared to other RAS superfamily members, such as the Rho family of small GTPases, which is well studied in intestinal homeostasis, the role of RAS GTPase signaling in maintaining tissue barrier integrity is not well established yet. Nevertheless, there is evidence that members of the RAS subfamily, such as (K)RAS, are important signaling molecules of epithelial junctions [7]. Indeed, many RAS oncoprotein effectors are proposed to converge on output responses such as cell adhesion, cell junction, or barrier function, e.g. mediated by RAP signaling, RAC-PAK-RHO signaling, actin-nectin-cadherin signaling, etc. [13]. Overall, cell–cell junctions contribute to the intestinal homeostasis by promoting barrier integrity. Under physiological conditions, other families than the RAS subfamily of small GTPases are mainly responsible for direct maintenance of the barrier integrity with cell–cell junctions, yet, alterations in these junctions can lead to tissue abnormalities that can disrupt homeostasis and promote cancer.

### Hypoxia-driven epithelial homeostasis

Due to the specific physiological organization of the gastrointestinal tract, oxygen levels fluctuate along the crypt-villus axis [4]. In the colon, IECs experience a relatively low-oxygen tension (< 10 mm Hg), which is called “physiological hypoxia” [48, 49]. This environment depends on multiple factors such as blood exchange, oxygen demands, and metabolism. It favours the development of ISCs, the survival of commensal bacteria and controls the innate and adaptive immunity. At the cellular level, the response to hypoxia is mediated by the hypoxia-induced transcription factor hypoxia-inducible factor 1 (HIF-1) that itself regulates the expression of specific genes (Fig. 4c). HIF-1 is composed of two subunits, HIF-1α and HIF-1β, and can activate hypoxia-responsive element-dependent gene expression. Under normoxic conditions, HIF-1α is rapidly degraded, while under hypoxic conditions, HIF-1α is stabilized and then translocated into the nucleus where it can bind to the co-activators CREB-binding protein (CBP) and p300. The resulting complex can target specific genes involved in different biological pathways connected to low-oxygen levels. The most well-characterized targets are involved in the maintenance of ISCs [50], in metabolic reprogramming [51–53], in the regulation of oxygen supply, and in angiogenesis [54]. Different studies have shown that the activation of HIF-1 plays a major role in the maintenance of colon homeostasis [49–57]. First, Karhaussen and colleagues demonstrated that epithelial HIF-1 has a protective effect in murine colitis and the loss of HIF-1 results in the loss of barrier function during colitis in vivo in mice [49]. Cummins and colleagues also reported the protective role of HIF-1 in in vivo mice model of colonic inflammation by showing that the protection from colitis is associated with reduced apoptosis of colon epithelial cells when HIF-1 is activated [55]. Moreover, Sun and colleagues showed that IEC-derived HIF-1 contributes to the maintenance of mucosal homeostasis by inducing interleukin (IL-33) expression in IBD [56], and later the same group showed that IECs-derived HIF-1 is essential for the homeostasis of intestinal intraepithelial lymphocytes and intestinal microbiota [57]. Altogether these findings suggest that HIF-1 is essential not only for the IECs but also for the maintenance of the colon environmental homeostasis balancing the microbiota and the immune cells.

The PI3K-AKT and MAPK pathways have been described to regulate HIF-1α activity by controlling HIF-1α synthesis. These pathways can all signal through RAS (including KRAS) and can be activated by growth factors [58–60] (Fig. 4c). Therefore, the activation of extracellular-signal-regulated kinase (ERK) can induce the phosphorylation of multiple downstream partners. The last step of this cascade is the phosphorylation of the eukaryotic translation initiation factor 4E (eIF-4E) which regulates the protein synthesis of HIF-1 via the increase of HIF-1 mRNA translation [61, 62]. Moreover, ERK is involved in HIF-1 synthesis and the transcriptional activation through phosphorylation of the coactivator CBP/p300, which leads to an increase of the HIF-1α/p300 complex, hence stimulating the transcriptional activation function [61]. To summarise, in physiological context and under hypoxic conditions, HIF-1α is stabilised and this mechanism can trigger the expression of target genes to adapt to the new hypoxic environments and maintain intestinal homeostasis. However, the expression of HIF-1α can also be regulated independently of the oxygen level. Certain signaling pathways which require the activation of ERK to synthesise and stabilise HIF-1α can be stimulated thanks to the presence of growth factors that activate RAS.

### Role of the gut microbiota in intestinal homeostasis

The colon forms a dynamic and complex barrier that separates the host immunity from the lumen where the commensal flora resides. The human intestine is colonised by a dynamic and diverse population of more than 100 trillion bacterial cells which have developed a beneficial relationship with the host immune system [63]. This symbiosis has been implicated in multiple functions such as immunity, nervous system, metabolism, and colonisation resistance, all known to play a role in intestinal homeostasis [64, 65] (Fig. 4d). The beneficial effect of the microbiota depends on its composition and its dysbiosis (e.g. imbalance in function or structures of gut microbiota) can lead to human disorders and diseases. In fact, an increasing number of studies have shown that microbiota dysbiosis is likely related to metabolic and inflammatory diseases, such as obesity or IBD [66], which are enhanced by external factors (e.g. antibiotics, dietary components and/or stress). In the context of CRC, bacteria have been shown to play a role in cell signaling [67–69]. The host genetic variation (e.g. single nucleotide polymorphisms) as well as environmental factors (e.g. diet, stress) are known to impact the composition of the microbiome [70], although this interplay still awaits to be fully elucidated.

In a KRAS-specific context, the role of KRAS signaling on the microbiome, or vice versa, still need to be elucidated, even though, it is accepted that the exposure to bacterial can shape the development of CRC [71, 72] of which KRAS can be one major player. Nevertheless, in KRAS-driven CRCs, it has been shown that genotoxic stress and some other factors, including metabolites produced by the microbiota, can facilitate genetic and epigenetic changes leading to carcinogenesis [73].

### Role of the immune system and control of the gut inflammation in intestinal homeostasis

As described above, the colon is a unique part of the gastrointestinal tract, which is constantly exposed to antigens derived from food and from the microbiota. Antigens present in the lumen influence the development and the maturation of intestinal tissue, as well as immune cells by training the immune system to tolerate the commensal microbiome, through the intestinal barrier [74–76] (Fig. 4e). This tolerogenic response enables the maintenance of a homeostatic balance against commensal antigens while avoiding to trigger an inflammatory reaction [77]. First, there is the physical barrier formed by IECs with TJs and mucus secretion, which separates the microbiota from the immune cells. Further, there is a biological control that is carried out by the secretion of antibacterial molecules and an intense trafficking of immune cells, which is described as “physiological inflammation”. However, these mechanisms are not sufficient to provide full protection. For example, in case of a disruption of the microbiota symbiosis, or a damage of the intestinal barrier, or the presence of antigens in restricted sites [78], an inflammatory response can be triggered.

The innate immunity is the first line of defence in the body to recognise pathogens and maintain homeostasis. Toll-like receptors (TLRs) are key innate immune sensors of the microbiota and are expressed in IECs and immune cells (e.g. dendritic cells (DCs), macrophages). Their activation induces several downstream signaling cascades resulting in the production of cytokines among other genes involved in the resolution of inflammation. The main signaling pathway leads to the activation of the transcription factor nuclear factor-kappa B (NF-κB) and the MAPKs p38 and JNK, which results in an increased expression of many pro-inflammatory cytokines. Additionally, TLRs stimulate the activation of the adaptive immune system such as the regulations and maturation of DCs, and the proliferation and differentiation of lymphocytes. The inflammatory response is also modulated by several other key players of the immune system such as lymphocyte T regulators (LTregs), macrophages, and IL-10, which are all involved in the resolution of the inflammation. The anti-inflammatory cytokine, IL-10, produced by immune cells (i.e. DCs, macrophages, neutrophils) but also by epithelial cells in the intestine, play an important role in limiting host immune response to prevent intestinal damage due to the inflammation. Upon activation, IL-10 receptor signal through the Januse kinase (JAK)/signal transducer and activator of transcription (STAT) 3 pathway and activates specific anti-inflammatory genes to suppress cytokine production and maintain intestinal homeostasis. IL-10 is also known to maintain the expression of LTregs to decrease the activation of immune cells for resolving the inflammatory state [79, 80]. Impaired resolution of inflammation can cause diseases like Crohn’s disease or cancer. In particular, long-term intestinal inflammation has an increased risk of colitis-associated cancer, especially CRC [81]. Inflammatory mediators present in CRC such as IL-10, TGF-β, LTregs or tumor-associated macrophages are known to play a critical role in the initiation, maintenance, and development of CRC [82].

There is growing evidence that supports the idea of a tissue-based model of carcinogenesis [83]. This theory proposes that epithelial cells with driver mutations cause uncontrolled proliferation in a cell-autonomous way, but that the tumor-microenvironment (TME) is part of the carcinogenesis process by, among others, maintaining an inflammatory environment, favouring the evasion from the immune system. Indeed, oncogenic KRAS is proposed to play a central role in both processes: as a mediator of sustained proliferation (via the MAPK pathway) and as a mediator of immune modulatory effects (via the activation of STAT3, the production of IL-6, the activation of the NLRP3 inflammasome and the release of chemokines) [84]. In summary, the immune system contributes to intestinal homeostasis by maintaining a tight regulation of inflammation and the integrity of the intestinal barrier. Inflammation can be triggered by abnormal environmental factors, commensal microbiota and by the immune system itself. Long-term intestinal inflammation has an increased risk of colitis-associated cancer, especially CRC [81].

### The central role of the metabolism in intestinal homeostasis

IECs need energy (like all cells) to grow, occasionally to proliferate, to absorb and digest molecules and also to adapt their metabolic rate in response to environmental cues (e.g. signals for cell fates) and nutrient availability [85]. Under physiological conditions, cells produce energy through the catabolism of food, which is mainly composed of carbohydrates, proteins, and fats. They are then broken down into glucose, amino acids, and glycerol/fatty acids respectively, and converted to pyruvate and acetyl co-enzyme A (Co-A) (Fig. 4f). Co-A is an intermediate molecule metabolised through the tricarboxylic acid pathway into energy, in the form of adenosine triphosphate (ATP) during oxidative phosphorylation (OXPHOS) into mitochondrial respiration. All these metabolic pathways are carried out in the presence of oxygen, however, if the oxygen level is low or absent, the cells will then use anaerobic respiration. In this case, the pyruvate will then be transformed into lactic acid, in a process called anaerobic glycolysis. This ability of cells to metabolize pyruvate to lactate in the presence or absence of oxygen is known as the Warburg effect [86]. It is often associated with proliferating cells as well as cancer cells, although it does not seem to have the same goal in both cases [87].

Regarding IECs, it has been shown that the energy metabolism along the crypt-villus axis in the small intestine is changing [66]. This allows us to speculate that depending on the anatomical localisation and nutrient availability in the intestine, cells do not have the same metabolic program. Indeed, it seems to be correlated to the phenotypes, type, and differentiation state of cells, such as ISCs compared to enterocytes. In particular, ISCs are of interest (especially the Lgr5 + stem cells population in the crypt base), which appear to be continually proliferative [88]. In fact, most proliferative cells rely on aerobic glycolysis (Warburg effect) in contrast to differentiated cells which rely mainly on oxidative phosphorylation. There are two types of Lgr5 + ISCs, the quiescent cells and the proliferative that will give rise to progenitor cells that are being differentiated [89]. For example, the proliferative Lgr5 + SCs, in order to proliferate, are limiting OXPHOS by downregulating the mitochondrial pyruvate carrier (MPC), which prevent cells from achieving an efficient pyruvate uptake into the mitochondria [90] (Fig. 4f). Fan and colleagues also demonstrate that those same cells (Lgr5 + proliferative ISC) exhibited a Warburg-like metabolic profile in epithelial hypoxia [91]. In fact, based on the hypothesis that ISCs Lgr5 + are cells at the origin of CRC [2], and that multiple metabolic pathways for ISCs function during homeostasis and tumorigenesis have been identified [92], many features of stem cells metabolism are similar to the “ cancer stem cells” observed in cancer [93]. Studies suggest that the modulation of glucose determines ISC self-renewal and proliferation (probably being highly glycolytic) [90, 94].

Lipids and cholesterol are involved in the regulation of ISC activity [95] and proliferation [96]. Thus, it has been suggested that metabolic reprogramming could be defined as a driver of stemness (self-renewal and proliferation) and tumorigenesis [97]. Rewiring of cellular metabolism is one of the main hallmarks of most cancer cells, which allow energy production and biomass production for their sustained high rates of cell division [98]. Reprogramming metabolism has been shown to be associated with oncogenic mutations such as KRAS [99], but also with proliferation of non-transformed tissues. Indeed, metabolic changes can occur in cells that are induced to proliferate such as activated T lymphocytes or ISCs [100]. Highly proliferative cells, like ISCs, need to support high proliferation rates due to their role in the regeneration of the intestinal epithelium, and thus differ from quiescent cell metabolism whose metabolism is characterized by high glycolysis, lactate production, biosynthesis of lipids and other macromolecules.

Signaling pathways that regulate metabolism of proliferating cells require glucose as well as growth factors to activate (K)RAS and the MAPK pathways to trigger the proliferative/survival phenotype. However, KRAS is often mutated leading to its hyperactivation in cancer, thereby mediating activation of multiple effectors/pathways like the PI3K/AKT and MAPK pathways, which both, can increase glycolysis and are involved in lipid biosynthesis. In conclusion, as mucosal development is dominated by self-renewal, proliferation and differentiation of epithelial cells, metabolic pathways during differentiation need to be adapted to the situation and environment to maintain intestinal function and homeostasis. Therefore, energy homeostasis requires a constant coordination between nutrient availability, cell fates and the regulation of energy.

## Selected examples of KRAS oncogenic mutations impacting intestinal homeostasis

### CRC and hypoxia

**Fig. 5:**
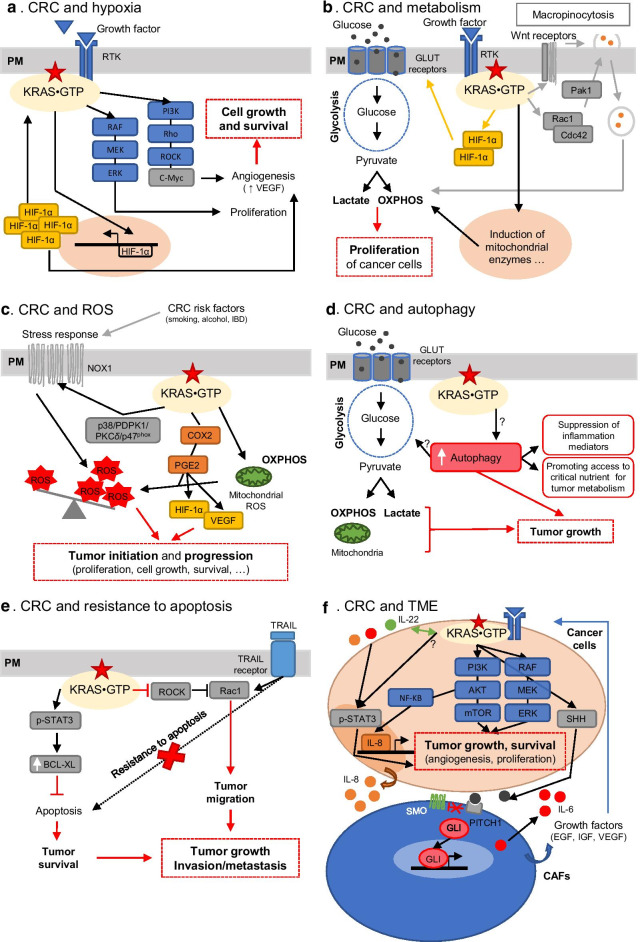
Schematic illustration of the impact of KRAS oncogenic mutations on intestinal homeostasis. **a** Oncogenic KRAS can regulate HIF-1α at the transcriptional level to induce multiple signaling processes including angiogenesis, but it can also regulate pro-angiogenic factors through the HIF-independent regulation of angiogenesis to enhance tumor growth. **b** Oncogenic KRAS supports cancer cells growth by enhancing metabolic pathways via transcriptional regulation of HIF-1α and macropinocytosis. **c** The accumulation of reactive oxygen species (ROS) in KRAS-driven cancer favours cancer initiation and progression through different pathways. **d** Autophagy in oncogenic KRAS-driven CRC supports tumor growth by increasing glucose metabolism, favouring access to nutrients, and decreasing inflammatory mediators. **e** Oncogenic KRAS confers apoptosis resistance through the up-regulation of Bcl-xL but also through TRAIL signaling rewiring. **f** The colon tumor microenvironment (TME) and oncogenic KRAS work together to promote tumor progression through, e.g. cancer-associated fibroblast (CAFs) as well as cytokines production and regulation

The colon is already exposed to “physiological hypoxia” when a tumor starts to increase in size, as such it creates an increasingly hypoxic environment, where nutrients and oxygen supply become limited [101]. Hypoxic conditions put cancer cells under selective pressure. In response to cellular adaptation, the angiogenesis is stimulated via the induction of vascular endothelial growth factor (VEGF) and HIF-1α is known to be a key mediator of this process, which enhances the survival of the tumor under hypoxia (Fig. 5a). The hypoxic tumor environment together with the ability of cancer cells to survive has been suggested to be associated with drug resistance to therapy [102], poor prognosis and several signaling pathway activations such as PI3K/AKT, MAPK and NOTCH signaling pathways [103, 104]. Each have been linked to KRAS, suggesting that KRAS plays a key role in hypoxia in cancer. Indeed, it has been demonstrated that KRAS oncogenic (especially KRAS^G12V^) can induce/up-regulate VEGF in hypoxia via HIF-1-independent mechanisms in colon cancer cells [105]. Signaling through the activation of PI3K/Rho/ROCK and c-myc signaling is an alternative to HIF-1α for the induction of VEGF and oncogenic KRAS can further enhance signaling through these pathways in CRC [106]. However, HIF-1α can also be directly regulated by oncogenic KRAS. As a matter of fact, HIF-1α is induced by KRAS^G12V^ at transcription level and overexpression of HIF-1α under hypoxia could increase KRAS^G12V^ activity and triggers its downstream signaling in colon cancer cells through a positive feedback loop [107]. Knowing the proliferation role of KRAS^G12V^ as well as hypoxia-induced resistance to therapy, after inhibition of EGF receptors (KRAS pathway) and HIF-1 s, a decrease in proliferation was observed. Therefore, the two signals seem to work together to promote cell proliferation and enable cancer cells to escape from EGFR-inhibitor treatment. Another study contradicts these results and shows that hypoxia increases the activity of KRAS^WT^ but not KRAS^MUT^ [108]. This divergence could be explained by the processing time to simulate hypoxia in the cells, 4 h [108] compared to 24 h [107] and also by an adaptation mechanism which would depend on the period of time required to induce/increase KRAS^G12V^ activity. Experiments carried out in colon cancer cell lines (DLD1 and HCT-116) mutated for KRAS have shown that a knock-out or -down of KRAS^MUT^ cells impaired the hypoxic induction of HIF-1α. In addition, it has been shown that oncogenic KRAS is capable of regulating HIF-1α and not HIF-2α at the level of translation [109].

To summarise, oncogenic KRAS in CRC can regulate HIF-1α at the translational level but is also able to regulate angiogenesis via the HIF-1-independent regulation of angiogenesis by enhancing the PI3K-Rho-ROCK and c-myc signaling induced hypoxia. KRAS mutants in CRC play a role via hypoxia to enhance the accessibility of nutrients for cancer cells (tumor) by creating new vessels.

### CRC and metabolism

Oncogenic KRAS is involved in mitochondrial metabolism through HIF-1α, as demonstrated in human colon cancer cells [110]. In line with the hallmarks of cancer described by Hanahan and Weinberg [111], RAS transformed cells undergo significant metabolic adaptations [112]. Chun and colleagues [110] showed that, as described previously, HIF-1α is activated in cancers due to the dysregulation of RAS signaling. They also showed that the induction of HIF-1α induces the expression of glucose transporter and glycolytic enzymes in hypoxic and normoxic conditions [113, 114] that promote glucose uptake and glycolysis and enhance the proliferation of colon cancer cells [115] (Fig. 5b). Moreover, Chun and colleagues suggest that CRC (HCT116) cells with oncogenic KRAS mutations and expressing HIF-1α can maintain ATP production (increasing mitochondrial respiration efficiency) and decrease or prevent toxic reactive oxygen species (ROS) generation (both via the regulation of the exchange of cyclooxygenase (COX) 4 and also via the induction of enzymes important for mitochondrial cardiolipin synthesis) [110]. ROS as well as ATP production seem to contribute to carcinogenesis through the induction of HIF-1α in glycolysis and aerobic respiration via oncogenic KRAS in CRC context.

To support cancer cell growth, particularly the needs for increased biomass, RAS-driven cancers are using macropinocytosis [116]. This mechanism allows cancer cells to recover materials from their surrounding/extracellular environment (e.g. fatty acids, glutamine, amino acids). Macropinocytosis stimulation can occur in the context of RAS transformation and can be enhanced by growth factors stimulation via the activation of ras-related C3 botulinum toxin substrate 1 (Rac1) and Cdc42 followed by the stimulation of p21-activated kinase 1 (Pak1) to induce actin polymerization which leads to increased membrane ruffling and macropinocytosis [117] (Fig. 5b). First, macropinocytosis has been shown in human pancreatic tumors, a cancer which features a near-universal mutation in KRAS [118]. Further, it has been demonstrated that oncogenic KRAS can mediate activation of canonical Wnt signaling to support tumor growth by promoting macropinocytosis [119], along with the induction of metabolic reprogramming (Warburg effect). It is interesting to mention that intestinal stem cells at the bottom of the crypt harbor a high glycolytic metabolism and high Wnt signaling levels [120], which are both involved in cancer hallmarks. Taken together, these studies support that oncogenic KRAS, as well as Wnt signalling are contributing to macropinocytosis in CRC.

### CRC and ROS

ROS are natural products formed by the partial reduction of oxygen such as superoxide anion (O2 −), hydrogen peroxide or hydroxyl radicals (HO) or nitric oxide (NO) [121]. ROS have important roles in cell physiology (signaling) to maintain homeostasis, all at low concentration. Different levels of ROS can induce various cell fates. Low levels are able to induce proliferation, differentiation and stress response activation, whereas high levels lead to DNA, protein and lipid damage which is able to result in senescence, cell death, malignant transformation or metastasis [122, 123]. Two varieties of ROS are distinguished. There is an exogenous source of ROS such as ultraviolet exposure and an endogenous source of ROS which generates cellular ROS as in the process of mitochondrial respiration (oxidative phosphorylation) or the nicotinamide adenine dinucleotide phosphate (NADPH) oxidase (NOX), which is mainly a response to stress. Under pathological conditions, high concentration of ROS is cytotoxic due to its deleterious potential to create damage as a result of oxidative stress occurring when cells can no longer produce an effective antioxidant response. This unbalanced situation can promote different types of diseases such as cancers including CRC [124]. Indeed, many risk factors associated with CRC are known to increase ROS generation such as smoking, alcohol consumption, or IBD [125], but it also has been shown that the oxidation process begins to develop in the polyps stage of colorectal adenocarcinoma [126] (reviewed in [127]).

The accumulation of ROS is understood as an essential mediator of RAS-induced transformation and tumorigenesis [128]. More recently, Lim and colleagues reviewed the roles of oncogenic RAS in ROS generation (on redox balance) with signaling pathways and mechanism driving oncogenic (K)RAS induction of cellular prooxidant and antioxidant programs for cancer development [129]. The authors proposed a model in which the cellular redox balance/homeostasis is linked to mutant RAS-mediated tumor initiation and progression. During tumor transformation and initiation RAS mutants can activate antioxidant programs to adapt to the high ROS production, leading to proliferation, and transformation (Fig. 5c). Additionally, mutant RAS during tumor progression can promote prooxidant programs too, which contributes to genetic instability, differentiation, or proliferation.

In CRC, apart from the oxidation of EGFR by ROS (EGFR cys797), ROS can also oxidize other components of the MAPK pathway. ROS can also be produced by specialized enzymes, the NADPH or NOX1 oxidases of the NOX family, located at the PM (Fig. 5c). High expression along epithelial surfaces exposed to the external environment was found, where NOX1 is located on apical cell surfaces and released ROS into extracellular environments. Oxidases are also induced by immune cytokines (e.g. interferon gamma (IFNγ), IL-4 and 13). It has been shown that NOX1 is expressed in normal colon epithelial cells and in CRC cells and that NOX-generated ROS can induce the activation of RAS by S-glutathionylation on cys118 [130]. Moreover, after inhibition of NOX1 expression, the production of superoxide is strongly attenuated and prevents oncogenic RAS-transformed phenotypes, such as anchorage-independent growth and morphological changes [105]. Another study suggested that NOX1 (over)expression was correlated with KRAS mutations (G12Cys, G12Asp, G13Asp, G12Val, G60Gly, Q61Lys, Q61His) in human colon tumors and in KRAS^G12V^ transgenic mice in the intestine compared to adjacent normal colon tissue [131]. These studies suggest that NOX1 plays an important role in CRCs and indeed it has been shown that the expression of NOX1 induces mitogenesis as well as angiogenesis, inhibition of apoptosis. Therefore, aberrant expression of NOX1 could contribute to the development of CRC enhanced by KRAS mutations. In support of the previous assumption, it has been shown that oncogenic KRAS promotes ROS generation in colon cancer cells (HCT116 KRAS^G13D^ and SW480 KRAS^G12V^) through the signalling cascade p38/PDPK1/PKC*δ*/p47^phox^/NOX1 [132].

Another important source of ROS in the colon are cyclooxygenases, in particular COX-2. Evidence supports a critical role for COX-2 during colorectal tumorigenesis [133–135] due to its role in producing prostaglandin E2 (PGE_2_). COX-2 is an enzyme that releases PGE_2_ and produces ROS (e.g. H_2_O_2_) [136]. It is an early response to inflammation induced by pro-inflammatory cytokines (e.g. IL-1α/b, IFNγ and tumor necrosis factor (TNF)-α) or KRAS oncogenic proteins [137, 138]. Thus, mutation resulting in aberrant RAS signaling have been implicated in COX-2 up-regulation as well as hypoxia through HIF (Fig. 5c). Indeed, KRAS can regulate COX-2 specifically and an over-expression of COX-2 is linked to an increased production of PGE_2_ that mediates cell proliferation. Studies have shown that KRAS^G12V^-induced ROS generation led to a significant increase in DNA single-strand breaks in a COX-2-dependent manner in mouse peripheral lung epithelial cells (E10 cells). It is particularly of interest because aberrant expressions of COX-2 in CRC seem to play an important role during CRC development [127, 139] by promoting cell growth and survival. Moreover, it has been shown that PGE_2_ enhances intestinal adenoma growth via the activation of the RAS-MAPK cascade in mice [140] in the same manner that constitutively active RAS does in murine intestinal adenoma. In addition, during hypoxia, COX-2 up-regulation results in a high level of PGE_2_ which promotes CRC cell survival via oncogenic RAS. However, PGE_2_ also enhances HIF-1 transcriptional activity and VEGF induction (angiogenesis) under hypoxic conditions [141] and normoxic conditions due to the activation of HIF-1α by oncogenic KRAS. Thus, the activation of oncogenic KRAS pathway is acting as a positive feedback loop to maintain an active pro-survival COX-2/PGE_2_ pathway during hostile microenvironmental conditions [141].

### CRC and autophagy

Autophagy is an important transformational mechanism in CRC that occurs when a colon cell shifts from normal to malignant. However, autophagy has a dual and contradictory role. On the one hand, in physiological context, autophagy helps to protect damaged cells and act as a surveillance mechanism [142]. On the other hand, it has been shown that autophagy supports tumor formation and that the survival of RAS-driven cancer cells requires autophagy by promoting access to nutrients that are critical to metabolism and tumor growth [143]. During the early stages of CRC, it has been reported that autophagy functions as a suppressor, compared to the later stages, where autophagy seems to act as a promoting factor [144]. Moreover, Guo and colleagues showed that activated RAS requires autophagy to maintain oxidative metabolism and tumorigenesis [145]. The authors showed that the expression of KRAS^G12V^ up-regulates basal autophagy and that, after down-regulation of essential autophagy proteins, tumor growth was impaired (as well as energy metabolism) in different human cancer cell lines bearing different RAS mutations such as the HCT116 colorectal carcinoma cell lines which expresses the KRAS^G13D^ mutation (Fig. 5d).

Deficiency in autophagy can cause impairment in tumor growth but also deregulation of amino acid levels and depletion of key substrates in mitochondrial metabolism which leads to abnormality in mitochondrial respiration mostly found in RAS-driven lung cancer [145–147]. In addition, it has been demonstrated that autophagy can remodel the majority of the cellular proteome for cell survival [148] (Fig. 5d). Indeed, autophagy is a selective mechanism that can target specifically critical signaling pathways involved in RAS-driven cancer to preserve cellular survival function. Moreover, the authors also suggested that autophagy may suppress the anti-tumor immune response in (K)RAS-driven cancer (HRAS^G12V^ and KRAS^G12D^) by an autophagic degradation of inflammation mediators. This mechanism could partly explain how autophagy supports RAS-driven tumor growth. Another aspect has been proposed by Lock and colleagues who demonstrated a connection between autophagy and glucose metabolism which seems to be driven by oncogenic KRAS [149]. They showed that glycolysis is increased in autophagy-competent cells versus autophagy-deficient cells harbouring KRAS mutations and that this process facilitated RAS-mediated adhesion-independent transformation in RAS-driven tumor growth. This observation is in line with the well-known Warburg effect or metabolic shift found in tumor cells where critical components of glycolysis are upregulated, resulting in an enhanced glucose uptake and higher glycolysis rates). In conclusion, autophagy is required for robust KRAS-driven CRC transformation by supporting tumor growth through increased glucose metabolism that specifically targets survival pathways.

### CRC and resistance to apoptosis: increase of anti-apoptotic B-cell lymphoma-extra-large (BCL-XL)

In physiological context, programmed cell death or apoptosis, serves as a security mechanism against tumorigenesis. Cancer cells develop mechanisms to escape apoptosis, such as deficiency in pro-apoptotic proteins (regulation, degradation) or overexpression of anti-apoptotic proteins, as well as p53 mutations or caspase activations, which results in therapy resistance in colon cancer [150]. KRAS mutant proteins in CRC have been associated with decreased apoptosis and treatment resistance due to defective apoptotic signaling and general regulation of these signals. For example, the level of BCL-XL protein, an anti-apoptotic protein, has been shown to be higher in KRAS^MUT^ tumors compared to KRAS^WT^ tumors [151]. Human cancer cells are commonly resistant to apoptosis due to the overexpression of anti-apoptotic Bcl-2 family (e.g. Bcl-2, Mcl-1 and Bcl-xL) or alternatively, due to down-regulation of pro-apoptotic BH3-only proteins (e.g. Noxa, Bik or Puma) [152]. This phenomenon could be explained by an increase in proteasomal activity [153] which in the homeostatic normal state is essential for the degradation and regulation of proteins. In cancers, proteasomal activity targets proteins which are involved in apoptosis and cell cycle regulation as well as in tumor progression in order to degrade them and escape cell death at the same time [154]. Indeed, KRAS^MUT^ can upregulate anti apoptotic Bcl-xL expression in CRC cell lines (KRAS^G12V^ and KRAS^G13D^) with a suggested role of ERK [155] or signal transducer and activators of transcription 3 (STAT3) transcription factor [156] in mediating Bcl-xL upregulation by KRAS^MUT^. KRAS^MUT^ can activate p-STAT3 (Tyr^705^) in the absence of interleukin-6 (IL-6) secretion which is normally a known regulator of STAT3, in order to upregulate Bcl-xL (transcriptional level) and confers apoptosis resistance in an IL-6 independent manner [156] (Fig. 5e).

### CRC and resistance to apoptosis: induction of tumor necrosis factor-related-inducing ligand (TRAIL)-induced apoptosis

The cytokine TNF-related apoptosis-inducing ligand (TRAIL or Apo2L) and CD95L/FasL are involved in the initiation of apoptosis through the activation of their death receptors (TRAIL-R1 (DR4) and TRAIL-R2 (DR5)), TNF-R1, and CD95 (Fas/APO1). Binding of TRAIL triggers receptors and leads to the activation of caspases cascades, resulting in apoptotic cell death [157]. TRAIL can selectively induce apoptosis in cancer cells in vivo but not in normal cells [158, 159]. As a result, the development of TRAIL-based cancer therapy has been promoted. However, many tumor cells are resistant to TRAIL-induced apoptosis, and TRAIL–TRAIL-R binding can also induce non-apoptotic signalling via activation of nuclear factor-κB (NF-κB), p38, ERK, SRC and RAC1 [160] (Fig. 5e).

In normal colon mucosa TRAIL and TRAIL-R2 are expressed mostly at the top of the epithelium, whereas TRAIL-R1 is detected all along the crypt axis. In carcinoma, TRAIL and TRAIL receptor expression are variable and did not correlate with disease-free survival [161]. However, KRAS mutations render CRC cells more resistant to CD95L/FasL (ligand of TNF receptor family such as TRAIL) and TRAIL-induced apoptosis, but also convert the respective ligand-induced signals into a migration-activating signal [162]. In addition to CRC, it has been demonstrated in non-small-cell lung carcinoma and pancreatic ductal that human receptor TRAIL-R2 is able to promote tumor growth, migration, invasion and metastasis. In particular, it was shown that the endogenous expression of the equivalent receptor in mice (mTRAIL-R) promotes KRAS-driven growth and metastasis by activating the small GTPase Rac1 in vivo [160]. Later, the same group shed light on Rac1 and KRAS involvement. They showed that KRAS is involved in the induction of migration through the TRAIL-TRAIL-R2 signaling pathway. However, this same pathway leads to autocrine stimulation of Rac-1-independent migration. Generally, the Rac-1 activity is inhibited by Rho-associated protein kinase (ROCK). However, oncogenic KRAS inhibits ROCK, thereby releasing Rac-1 to be fully activated by TRAIL-R2 and induce migration instead of apoptosis [160, 163].

### CRC and the TME

Cancer cells are subjected to a constant assault of signals that comes from the tumor itself but also from its surrounding environment. Indeed, the TME is crucial for cancer progression, aggressiveness, changes in tumor cells expression profile, escaping from the immune system or maintaining tumor-promoting inflammation [84]. The TME is mainly composed of different cell types such as immune cells, cancer-associated fibroblast (CAFs), cancer associated macrophages (mostly in M2 phenotype; [164]), adipocytes and pericytes but also non cellular components such as the extracellular matrix (ECM) and cytokines. All components of the TME form a dynamic environment and cooperate and communicate together with tumor cells to promote cancer progression. Targeting the TME is currently a promising path which aims to suppress the support of the TME to the tumor cell with the hope of enhancing an immune response or chemotherapy against the tumor. It is important to keep in mind that all components of the TME are interconnected and regulate each other’s properties. Therefore, we can speculate that by affecting one of the components, KRAS^MUT^ cancer cells are likely affecting the entire TME behaviour. The TME is regulating CRC cells/tumors but KRAS^MUT^ tumors also are involved in the recruitment of specific populations [165].

*CAFs in TME*. Among TME components, CAFs are the most dominant cellular constituents of the stroma and are linked with primary and metastatic CRC [166, 167]. Due to their plasticity, CAFs can be activated through the interaction with tumor cells and are thereby able to enhance tumor initiation and progression, migration, invasion, and are modulators of the immune system as well as regulators of ECM remodelling, and angiogenesis [168, 169]. Thus cancer cells can modulate the properties of fibroblasts and influence the TME. In CRC context, a crosstalk between KRAS^MUT^ cells and CAFs has been suggested [170]. Moreover, overexpression of fibroblast activation protein (FAB), a surface glycoprotein expressed almost exclusively expressed on CAFs, has been shown to promote growth in animal models [171].

Despite the lack of direct evidence that CAFs can be associated with oncogenic KRAS, a study found a correlation between somatic mutations and CAF markers [172]. The authors demonstrated differential expression levels of ACTA2 (CAFs marker) among tumors with different KRAS mutation status suggesting that CAFs may play a role in selecting tumor cells with specific driver mutations [172]. To support this hypothesis, it was demonstrated that colon CAFs can secrete many growth factors including EGF, HGF, insulin growth factor (IGF)1/2, PGE-2, PDGF, FGF-1 and VEGF [173, 174]. All these factors act through the activation of the MAPK and PI3K/AKT pathways downstream of KRAS, and promote proliferation, cell survival, protein synthesis, cytoskeletal rearrangements or invasion and which could be enhanced in the presence of KRAS^MUT^.

CAFs are also known to secrete cytokines, including IL-6, which is a molecule found to be significantly elevated in CRC tissues [175–177] (Fig. 5f). The production of IL-6 in fibroblasts has been shown to be regulated by KRAS in a paracrine fashion in pancreatic cancer cells. The induction of sonic hedgehog (SHH) by KRAS in cancer cells triggers the expression of the transcription factor GLI1 in fibroblasts [178, 179]. As a result, IL-6 is produced and secreted into the TME. The loss of GLI1 has been shown to impair KRAS-induced pancreatic carcinogenesis. IL-6 can also be produced directly by cancer cells, such as in basal cell carcinoma (nonmelanoma skin cancer) where IL-6 is secreted through the HH/ GLI1 pathway via STAT3 activating IL-6 [180]. Thus, it can be speculated that the mechanism described above may be similar in KRAS-driven CRC, especially because almost all pancreatic ductal adenocarcinoma harbour mutations within the *KRAS* gene. Nevertheless, HH signaling is not fully understood in CRC and the potential benefits of IL-6 inhibition in CRC are still unknown [181].

It has been suggested that oncogenic signals such as KRAS may affect HH signaling because both aberrant activation of HH signaling and RAS mutations, are found in colon cancers [182] (Fig. 5f). Briefly, the canonical activation of HH-GLI pathway occurs through binding of HH ligands to the protein patched homolog 1 (PITCH1) receptor, which derepresses the SMO protein which leads to the final effectors: GLI transcription factor activation of the HH-GLI pathway. In contrast, the non-canonical ways of GLI activation occurring in cancers are SMO-independent. Indeed, in CRC, previous attempts to block HH signaling at the level of SMO, induced only moderate cytotoxicity [183], compared to the inhibition of GLI directly in human colon cancer cells [184]. Moreover, the HH signaling is activated in CRC by ligand-dependent mechanisms with overexpression of SHH [185, 186], as well as the SHH-GLI1 pathway [187] but both pathways, canonical and non-canonical can co-exist in cancer context. In addition, GLI1 activity can be enhanced in a dose-dependent manner in different colorectal cancer cells (KRAS^G12V^ luciferase reporter) by RAS, mitogen-activated protein kinase kinase (MEK) and AKT [188]. Although, GLI1 could be enhanced indirectly through the upregulation of β-catenin by oncogenic KRAS and loss of p53 as well as inactivation of PTEN [189, 190]. In summary, GLI1 activity is boosted by oncogenes, such as KRAS and loss of tumor suppressors (p53, PTEN) which are all characteristics of CRC progression. In addition, it has also been suggested that multiple crosstalk points are possible between both the Wnt/β-catenin and Hedgehog/Gli signaling pathways in colon cancer [191]. Both pathways can be promoted/enhanced by oncogenic KRAS due to the fact that KRAS^MUT^ has been shown to activate the Wnt/β-catenin pathway in the context of intestinal tumor formation and progression [189].

*Immune infiltrations and cytokines production in CRC TME*. Positive immune infiltration used as a prognostic value in CRC has been first demonstrated with tumor-infiltrating lymphocytes [192]. Indeed, the anti-tumoral immune surveillance is realised by several players. Here, we focus on lymphocytes and IFNγ, which are both promoting the host response to primary tumors. In CRC context, some studies demonstrated that oncogenic KRAS is able to reduce the expression of IFNγ targeted genes in KRAS^MUT^ tumor cells (HCT-116 cells with KRAS^G13D^), including STAT1 and MHC-II. STAT1 expression is stimulated by IFNγ and leads to the expression of MHC-II at the tumor cells surface which are molecules presenting tumoral antigens to the host immune system. The downregulation of this pathway (IFNγ-STAT1-MHC-II) drastically reduces the ability of tumoral cells to stimulate lymphocytes to induce cell death. As a consequence, it is also promoting tumoral immune evasion [193–195]. Additionally, the same mechanism has been reported by Lal and colleagues in RAS mutant CRC cells, where KRAS mutations were shown to strongly impact tumor immune infiltration compared to KRAS^WT^ samples [195]. The authors observed that samples with KRAS mutations were associated with a down regulation of the IFNγ pathway and with a reduced infiltration of Th1/cytotoxic T cells immunity in CRC. This is consistent with the CMS where KRAS^MUT^ tumors are described as poorly immunogenic [23].

Among the cytokines detected in CRC, here we are focusing on those having a link or correlation with KRAS-driven CRC. To note, not only tumor cells produce cytokines to maintain a pro-inflammatory TME, but also stromal cells of the TME can release cytokines which in turn can act on CRC tumor cells to secrete cytokines and favour a TME. CRC KRAS mutated cells show a higher expression at basal level of pro-angiogenic chemokines such as CXCL1 and 8, and a low basal expression of inflammatory cytokines compared to KRAS^WT^ cells [196]. Furthermore, IL-22 has been shown to promote tumor progression in murine models of CRC [197–199] and to play a role in promoting CRC stemness [200]. A link between IL-22 signaling and KRAS-driven CRC has been demonstrated [201]. The authors suggested that IL-22 and KRAS^MUT^ cooperatively enhance proliferation. This could be explained, in part, by an augmentation of the Myc pathway and its targeted genes, which are known to be involved in cell proliferation, metabolism, and cellular growth. Another interesting interleukin, IL-6, because of its similarity with IL-22 can trigger the expression of STAT3 (cytokine production) and is a key regulator of CRC development [202].

Another cytokine, granulocyte–macrophage colony-stimulating factor (GM-CSF), has been shown to be upregulated in CRC patients and in particular in those harbouring KRAS^MUT^ [203]. Additionally, in CRC context, it has been demonstrated that KRASG12D was able to suppress the expression of interferon regulatory factor 2 (IRF2), which is a negative regulator of the CXCL3 chemokine [204]. CXCL3 facilitates the recruitment of activated T cells, tumor-associated macrophages (TAMs) as well as myeloid-derived suppressor cells (MDSCs) among others. The authors have further shown that this mechanism leads to a decreased response to immune checkpoint blockade (ICB) with anti-programmed death (PD-1). IL-17 expression levels were also higher in KRAS^MUT^ positive CRC tissue [203]. IL-17 is known to be secreted by T-helper cell 17 (Th17), a pro-inflammatory subset of Th cells, which were shown, to accelerate tumor formation in lung cancers [205]. Moreover, human CRC also exhibits up-regulation of IL-23 [206], a pro-inflammatory cytokine. IL-23 is known for enhancing the expression of Th17 cells, inducing immune cell activation aggravates gut inflammation and promoting CRC tumor growth and progression via Th17 cells [206, 207]. Some cytokines are also involved in the regulation of inflammation that are promoting angiogenesis (stromal response). For example, RAS-induced IL-8 secretion has been shown to play a critical role in tumor growth and angiogenesis (neovascularization) by being a transcriptional target of RAS signaling (including constitutively active KRAS^G12V^) [208].

## Conclusions

KRAS is a critical signaling protein that is central to both, the maintenance of intestinal homeostasis and CRC development and progression. However, despite multiple evidences that KRAS is involved in those (patho)physiological processes, the actual downstream networks and pathway are still not clearly defined. Network-centric approaches to drug development and cancer treatment have become essential in recent years. The mapping of protein–protein interaction networks is a powerful tool to study molecular mechanisms of signal transduction and its aberrations in diseases, such as cancer [209–211]. However, until now most of the interaction screenings have been performed in cell lines that are limited in terms of capturing network functions arising from the biological context of a three-dimensional tissue organisation [211, 212]. Henceforth, to understand context-specific network rewiring and, importantly, link network states to specific phenotypes, will require mapping of (KRAS-mediated) cellular networks in (patho)physiologically relevant model systems (e.g. transwell and co-culture systems). This will enable to experimentally mimic different microenvironmental and TME contexts. Further, this should include a basic change in paradigm, from cells being cultured in non-physiological conditions, such as high glucose and high oxygen, to more physiologically relevant growth conditions.

Cancer is a global health burden [213] and indeed the combat against cancer is one of the EU research priorities (“EU cancer mission”; https://ec.europa.eu/info/horizon-europe/missions-horizon-europe/cancer_en). CRC is one of the most frequent cancers, and in particular for KRAS mutant CRC no targeted therapies are available that can successfully treat this type of tumor. KRAS itself remains undruggable despite advances in inhibitors for the KRAS^G12C^ mutation that demonstrated anti-tumor activity in clinical trials [214]. Therefore, understanding networks downstream of KRAS and how they link to the cancer phenotype is a key future goal. Still, the immense obstacle of tumor heterogeneity among CRC patients persists and, even with current subtyping of CRC, it is likely that no drug regime will work for a specific CRC subtype [215, 216]. Indeed, personalized medicine approaches for CRC that build on multiple ‘omics’ data and the use of patient-obtained biopsies are a promising way forward [215, 216]. As a major step forward, recently a personalized medicine pipeline was developed, where patient-matched cell lines were used to identify new therapies and pathways to treat metastatic CRC [217].

Within the personalized medicine approach, another important path to pursue is incorporation of quantitative data (e.g. quantitative proteomics; [218]) and the development of computational predictive models [219]. Those models are expected to not only help to better understand the underlying cancer biology, but to greatly assist in predicting tumour responses, toxicity, and the optimal drug treatment regime. Of note, already in 1865 Claude Bernard formulated his vision of quantitative biology [220]: “*Although the application of mathematics to every aspect of science is its ultimate goal, biology is still too complex and poorly understood. Therefore, for now the goal of medical science should be to discover all the new facts possible. Qualitative analysis must always precede quantitative analysis*.” 150 year later it is clear that we have accumulated sufficient qualitative data to now proceed to the next step: the acquisition of quantitative data in (patho) physiological relevant systems and the generation of computational models for cell signaling networks. Ultimately, when applied to CRC biopsies, this is expected to enormously benefit patient treatment and survival.

## Data Availability

Not applicable.

## Abbreviations

AFDN: Afadin
AJ: Adhesion junction
APC: Adenomatous polyposis coli
ATP: Adenosine triphosphate
Bcl-xL: B-cell lymphoma-extra-large
CAF: Cancer-associated fibroblast
CBP: CREB-binding protein
CMS: Consensus molecular subtypes
Co-A: Co-enzyme A
COX: Cyclooxygenase
CRC: Colorectal cancer
DC: Dendritic cell
ECM: Extracellular matrix
EGF: Epidermal growth factor
EGFR: Epidermal growth factor receptor
eIF-4E: Eukaryotic translation initiation factor 4E
ERK: Extracellular-signal-regulated kinase
FAB: Fibroblast activation protein
Foxp3: Forkhead/winged-helix transcription factor box P3
GDP: Guanosine diphosphate
GEF: Guanine nucleotide exchange factor
GLUT: Glucose transporter
GM-CSF: Granulocyte-macrophage colony-stimulating factor
GTP: Guanosine triphosphate
HIF-1: Hypoxia-inducible factor-1
HH: Hedgehog
IBD: Inflammatory bowel disease
IEC: Intestinal epithelial cell
IGF: Insulin growth factor
IL: Interleukin
IFN: Interferon
ISC: Intestinal stem cell
Lgr5 +: Leucine rich G-proteins-coupled receptor 5
LTregs: Lymphocyte T regulators
MAPK: Mitogen-activated protein kinase
MDSC: Myeloid-derived suppressor cell
MEK: Mitogen-activated protein kinase kinase
MPC: Mitochondrial pyruvate carrier
NADPH: Nicotinamide adenine dinucleotide phosphate
NF-κB: Nuclear factor-kappa B
NO: Nitric oxide
NOX: Nicotinamide adenine dinucleotide phosphate oxidase
OXPHOS: Oxidative phosphorylation
Pak1: P21-activated kinase 1
PGE2: Prostaglandin E2
PI3K: Phosphoinositide 3-kinases
PITCH1: Protein patched homolog 1
PM: Plasma membrane
Rac1: Ras-related C3 botulinum toxin substrate 1
RBD: Ras binding domain
ROCK: Rho-associated protein kinase
ROS: Reactive oxygen species
SH2: Src homology 2
SHH: Sonic hedgehog
SMO: Smoothened frizzled class receptor
STAT: Signal transducer and activators of transcription
TAM: Tumor-associated macrophage
TCGA: The Cancer Genome Atlas
TLR: Toll-like receptor
Th1: T helper type 1
TJ: Tight junction
TME: Tumor microenvironement
TNF: Tumor necrosis factor
TRAIL: Tumor necrosis factor-related apoptosis-inducing ligand
Tregs: Regulatory T cells
VEGF: Vascular endothelial growth factor
Wnt: Wingless-related integration site

## References

[1] Allaire JM, Crowley SM, Law HT, Chang SY, Ko HJ, Vallance BA (2018). The intestinal epithelium: central coordinator of mucosal immunity. Trends Immunol.

[2] Baker AM, Cereser B, Melton S, Fletcher AG, Rodriguez-Justo M, Tadrous PJ, Humphries A, Elia G, McDonald SA, Wright NA (2014). Quantification of crypt and stem cell evolution in the normal and neoplastic human colon. Cell Rep.

[3] Buckley A, Turner JR (2018). Cell biology of tight junction barrier regulation and mucosal disease. Cold Spring Harb Perspect Biol.

[4] Zheng L, Kelly CJ, Colgan SP (2015). Physiologic hypoxia and oxygen homeostasis in the healthy intestine. A review in the theme: cellular responses to hypoxia. Am J Physiol Cell Physiol.

[5] De la Fuente M, MacDonald TT, Hermoso MA (2019). Editorial: intestinal homeostasis and disease. A complex partnership between immune cells, non-immune cells, and the microbiome. Front Immunol.

[6] Colicelli J (2004). Human RAS superfamily proteins and related GTPases. Sci STKE.

[7] Citalan-Madrid AF, Garcia-Ponce A, Vargas-Robles H, Betanzos A, Schnoor M (2013). Small GTPases of the Ras superfamily regulate intestinal epithelial homeostasis and barrier function via common and unique mechanisms. Tissue Barriers.

[8] Vetter IR, Wittinghofer A (2001). The guanine nucleotide-binding switch in three dimensions. Science.

[9] Simanshu DK, Nissley DV, McCormick F (2017). RAS proteins and their regulators in human disease. Cell.

[10] Karnoub AE, Weinberg RA (2008). Ras oncogenes: split personalities. Nat Rev Mol Cell Biol.

[11] Vasaikar S, Huang C, Wang X, Petyuk VA, Savage SR, Wen B, Dou Y, Zhang Y, Shi Z, Arshad OA (2019). Proteogenomic analysis of human colon cancer reveals new therapeutic opportunities. Cell.

[12] Wang D, Eraslan B, Wieland T, Hallstrom B, Hopf T, Zolg DP, Zecha J, Asplund A, Li LH, Meng C (2019). A deep proteome and transcriptome abundance atlas of 29 healthy human tissues. Mol Syst Biol.

[13] Ibanez Gaspar V, Catozzi S, Ternet C, Luthert PJ, Kiel C (2020). Analysis of Ras-effector interaction competition in large intestine and colorectal cancer context. Small GTPases.

[14] Spit M, Koo BK, Maurice MM (2018). Tales from the crypt: intestinal niche signals in tissue renewal, plasticity and cancer. Open Biol.

[15] Johansson J, Naszai M, Hodder MC, Pickering KA, Miller BW, Ridgway RA, Yu Y, Peschard P, Brachmann S, Campbell AD (2019). RAL GTPases drive intestinal stem cell function and regeneration through internalization of WNT Signalosomes. Cell Stem Cell.

[16] Hong AW, Meng Z, Guan KL (2016). The Hippo pathway in intestinal regeneration and disease. Nat Rev Gastroenterol Hepatol.

[17] Garcia MA, Nelson WJ, Chavez N (2018). Cell-cell junctions organize structural and signaling networks. Cold Spring Harb Perspect Biol.

[18] Wang W, Kandimalla R, Huang H, Zhu L, Li Y, Gao F, Goel A, Wang X (2019). Molecular subtyping of colorectal cancer: recent progress, new challenges and emerging opportunities. Semin Cancer Biol.

[19] Pino MS, Chung DC (2010). The chromosomal instability pathway in colon cancer. Gastroenterology.

[20] Nguyen HT, Duong HQ (2018). The molecular characteristics of colorectal cancer: implications for diagnosis and therapy. Oncol Lett.

[21] Yang L, Wang S, Lee JJ, Lee S, Lee E, Shinbrot E, Wheeler DA, Kucherlapati R, Park PJ (2019). An enhanced genetic model of colorectal cancer progression history. Genome Biol.

[22] Dekker E, Tanis PJ, Vleugels JLA, Kasi PM, Wallace MB (2019). Colorectal cancer. Lancet.

[23] Guinney J, Dienstmann R, Wang X, de Reynies A, Schlicker A, Soneson C, Marisa L, Roepman P, Nyamundanda G, Angelino P (2015). The consensus molecular subtypes of colorectal cancer. Nat Med.

[24] Dienstmann R, Connor K, Byrne AT, Consortium C (2020). Precision therapy in RAS mutant colorectal cancer. Gastroenterology.

[25] Ellis CA, Clark G (2000). The importance of being K-Ras. Cell Signal.

[26] Engin HB, Carlin D, Pratt D, Carter H (2017). Modeling of RAS complexes supports roles in cancer for less studied partners. BMC Biophys.

[27] Haigis KM (2017). KRAS alleles: the devil is in the detail. Trends Cancer.

[28] Imamura Y, Morikawa T, Liao X, Lochhead P, Kuchiba A, Yamauchi M, Qian ZR, Nishihara R, Meyerhardt JA, Haigis KM (2012). Specific mutations in KRAS codons 12 and 13, and patient prognosis in 1075 BRAF wild-type colorectal cancers. Clin Cancer Res.

[29] Prior IA, Lewis PD, Mattos C (2012). A comprehensive survey of Ras mutations in cancer. Cancer Res.

[30] Munoz-Maldonado C, Zimmer Y, Medova M (2019). A Comparative analysis of individual RAS mutations in cancer biology. Front Oncol.

[31] Catozzi S, Halasz, M, Kiel, C. Predicted ‘wiring landscape’ of Ras-effector interactions in 29 human tissues. NPJ Systems Biology and Applications in press. 10.1038/s41540-021-00170-0 PMC7881153 33580066

[32] Barker N, van Es JH, Kuipers J, Kujala P, van den Born M, Cozijnsen M, Haegebarth A, Korving J, Begthel H, Peters PJ (2007). Identification of stem cells in small intestine and colon by marker gene Lgr5. Nature.

[33] Takeda N, Jain R, LeBoeuf MR, Wang Q, Lu MM, Epstein JA (2011). Interconversion between intestinal stem cell populations in distinct niches. Science.

[34] Tian H, Biehs B, Warming S, Leong KG, Rangell L, Klein OD, de Sauvage FJ (2011). A reserve stem cell population in small intestine renders Lgr5-positive cells dispensable. Nature.

[35] Beumer J, Clevers H (2016). Regulation and plasticity of intestinal stem cells during homeostasis and regeneration. Development.

[36] Sato T, Stange DE, Ferrante M, Vries RG, Van Es JH, Van den Brink S, Van Houdt WJ, Pronk A, Van Gorp J, Siersema PD (2011). Long-term expansion of epithelial organoids from human colon, adenoma, adenocarcinoma, and Barrett's epithelium. Gastroenterology.

[37] Fearon ER, Vogelstein B (1990). A genetic model for colorectal tumorigenesis. Cell.

[38] Markowitz SD, Bertagnolli MM (2009). Molecular origins of cancer: Molecular basis of colorectal cancer. N Engl J Med.

[39] Barker N, Ridgway RA, van Es JH, van de Wetering M, Begthel H, van den Born M, Danenberg E, Clarke AR, Sansom OJ, Clevers H (2009). Crypt stem cells as the cells-of-origin of intestinal cancer. Nature.

[40] Dalerba P, Dylla SJ, Park IK, Liu R, Wang X, Cho RW, Hoey T, Gurney A, Huang EH, Simeone DM (2007). Phenotypic characterization of human colorectal cancer stem cells. Proc Natl Acad Sci U S A.

[41] Huels DJ, Sansom OJ (2015). Stem vs non-stem cell origin of colorectal cancer. Br J Cancer.

[42] Le Rolle AF, Chiu TK, Zeng Z, Shia J, Weiser MR, Paty PB, Chiu VK (2016). Oncogenic KRAS activates an embryonic stem cell-like program in human colon cancer initiation. Oncotarget.

[43] Moon BS, Jeong WJ, Park J, Kim TI, Min S, Choi KY (2014). Role of oncogenic K-Ras in cancer stem cell activation by aberrant Wnt/beta-catenin signaling. J Natl Cancer Inst.

[44] Helander HF, Fandriks L (2014). Surface area of the digestive tract—revisited. Scand J Gastroenterol.

[45] Zihni C, Mills C, Matter K, Balda MS (2016). Tight junctions: from simple barriers to multifunctional molecular gates. Nat Rev Mol Cell Biol.

[46] Citi S, Spadaro D, Schneider Y, Stutz J, Pulimeno P (2011). Regulation of small GTPases at epithelial cell-cell junctions. Mol Membr Biol.

[47] Young A, Lyons J, Miller AL, Phan VT, Alarcon IR, McCormick F (2009). Ras signaling and therapies. Adv Cancer Res.

[48] Albenberg L, Esipova TV, Judge CP, Bittinger K, Chen J, Laughlin A, Grunberg S, Baldassano RN, Lewis JD, Li H (2014). Correlation between intraluminal oxygen gradient and radial partitioning of intestinal microbiota. Gastroenterology.

[49] Karhausen J, Furuta GT, Tomaszewski JE, Johnson RS, Colgan SP, Haase VH (2004). Epithelial hypoxia-inducible factor-1 is protective in murine experimental colitis. J Clin Invest.

[50] Covello KL, Kehler J, Yu H, Gordan JD, Arsham AM, Hu CJ, Labosky PA, Simon MC, Keith B (2006). HIF-2alpha regulates Oct-4: effects of hypoxia on stem cell function, embryonic development, and tumor growth. Genes Dev.

[51] Dengler VL, Galbraith M, Espinosa JM (2014). Transcriptional regulation by hypoxia inducible factors. Crit Rev Biochem Mol Biol.

[52] Benita Y, Kikuchi H, Smith AD, Zhang MQ, Chung DC, Xavier RJ (2009). An integrative genomics approach identifies Hypoxia Inducible Factor-1 (HIF-1)-target genes that form the core response to hypoxia. Nucleic Acids Res.

[53] Semenza GL (2012). Hypoxia-inducible factors: mediators of cancer progression and targets for cancer therapy. Trends Pharmacol Sci.

[54] Feldser D, Agani F, Iyer NV, Pak B, Ferreira G, Semenza GL (1999). Reciprocal positive regulation of hypoxia-inducible factor 1alpha and insulin-like growth factor 2. Cancer Res.

[55] Cummins EP, Seeballuck F, Keely SJ, Mangan NE, Callanan JJ, Fallon PG, Taylor CT (2008). The hydroxylase inhibitor dimethyloxalylglycine is protective in a murine model of colitis. Gastroenterology.

[56] Sun L, Wang W, Xiao W, Yang H (2016). The roles of cathelicidin LL-37 in inflammatory bowel disease. Inflamm Bowel Dis.

[57] Sun L, Li T, Tang H, Yu K, Ma Y, Yu M, Qiu Y, Xu P, Xiao W, Yang H (2019). Intestinal epithelial cells-derived hypoxia-inducible factor-1alpha Is essential for the homeostasis of intestinal intraepithelial lymphocytes. Front Immunol.

[58] Fukuda R, Hirota K, Fan F, Jung YD, Ellis LM, Semenza GL (2002). Insulin-like growth factor 1 induces hypoxia-inducible factor 1-mediated vascular endothelial growth factor expression, which is dependent on MAP kinase and phosphatidylinositol 3-kinase signaling in colon cancer cells. J Biol Chem.

[59] Semenza GL (2003). Targeting HIF-1 for cancer therapy. Nat Rev Cancer.

[60] Lee JW, Bae SH, Jeong JW, Kim SH, Kim KW (2004). Hypoxia-inducible factor (HIF-1)alpha: its protein stability and biological functions. Exp Mol Med.

[61] Sang N, Stiehl DP, Bohensky J, Leshchinsky I, Srinivas V, Caro J (2003). MAPK signaling up-regulates the activity of hypoxia-inducible factors by its effects on p300. J Biol Chem.

[62] Masoud GN, Li W (2015). HIF-1alpha pathway: role, regulation and intervention for cancer therapy. Acta Pharm Sin B.

[63] Qin J, Li R, Raes J, Arumugam M, Burgdorf KS, Manichanh C, Nielsen T, Pons N, Levenez F, Yamada T (2010). A human gut microbial gene catalogue established by metagenomic sequencing. Nature.

[64] Wang W, Hu H, Zijlstra RT, Zheng J, Ganzle MG (2019). Metagenomic reconstructions of gut microbial metabolism in weanling pigs. Microbiome.

[65] Rajakovich LJ, Balskus EP (2019). Metabolic functions of the human gut microbiota: the role of metalloenzymes. Nat Prod Rep.

[66] Boulange CL, Neves AL, Chilloux J, Nicholson JK, Dumas ME (2016). Impact of the gut microbiota on inflammation, obesity, and metabolic disease. Genome Med.

[67] Burns MB, Lynch J, Starr TK, Knights D, Blekhman R (2015). Virulence genes are a signature of the microbiome in the colorectal tumor microenvironment. Genome Med.

[68] Flemer B, Lynch DB, Brown JM, Jeffery IB, Ryan FJ, Claesson MJ, O'Riordain M, Shanahan F, O'Toole PW (2017). Tumour-associated and non-tumour-associated microbiota in colorectal cancer. Gut.

[69] Rubinstein MR, Wang X, Liu W, Hao Y, Cai G, Han YW (2013). Fusobacterium nucleatum promotes colorectal carcinogenesis by modulating E-cadherin/beta-catenin signaling via its FadA adhesin. Cell Host Microbe.

[70] Blekhman R, Goodrich JK, Huang K, Sun Q, Bukowski R, Bell JT, Spector TD, Keinan A, Ley RE, Gevers D (2015). Host genetic variation impacts microbiome composition across human body sites. Genome Biol.

[71] Mima K, Sukawa Y, Nishihara R, Qian ZR, Yamauchi M, Inamura K, Kim SA, Masuda A, Nowak JA, Nosho K (2015). Fusobacterium nucleatum and T cells in colorectal carcinoma. JAMA Oncol.

[72] Zou S, Fang L, Lee MH (2018). Dysbiosis of gut microbiota in promoting the development of colorectal cancer. Gastroenterol Rep (Oxf).

[73] Guerriero JL (2019). Macrophages: their untold story in T cell activation and function. Int Rev Cell Mol Biol.

[74] Kamada N, Rogler G (2016). The innate immune system: a trigger for many chronic inflammatory intestinal diseases. Inflamm Intest Dis.

[75] Belkaid Y, Hand TW (2014). Role of the microbiota in immunity and inflammation. Cell.

[76] Ohland CL, Jobin C (2015). Microbial activities and intestinal homeostasis: A delicate balance between health and disease. Cell Mol Gastroenterol Hepatol.

[77] Peterson LW, Artis D (2014). Intestinal epithelial cells: regulators of barrier function and immune homeostasis. Nat Rev Immunol.

[78] Kayama H, Takeda K (2016). Functions of innate immune cells and commensal bacteria in gut homeostasis. J Biochem.

[79] Ouyang W, Rutz S, Crellin NK, Valdez PA, Hymowitz SG (2011). Regulation and functions of the IL-10 family of cytokines in inflammation and disease. Annu Rev Immunol.

[80] Hadis U, Wahl B, Schulz O, Hardtke-Wolenski M, Schippers A, Wagner N, Muller W, Sparwasser T, Forster R, Pabst O (2011). Intestinal tolerance requires gut homing and expansion of FoxP3+ regulatory T cells in the lamina propria. Immunity.

[81] Keller DS, Windsor A, Cohen R, Chand M (2019). Colorectal cancer in inflammatory bowel disease: review of the evidence. Tech Coloproctol.

[82] Mager LF, Wasmer MH, Rau TT, Krebs P (2016). Cytokine-induced modulation of colorectal cancer. Front Oncol.

[83] Ruckert F, Sticht C, Niedergethmann M (2012). Molecular mechanism of the "feedback loop" model of carcinogenesis. Commun Integr Biol.

[84] Hamarsheh S, Gross O, Brummer T, Zeiser R (2020). Immune modulatory effects of oncogenic KRAS in cancer. Nat Commun.

[85] Mihaylova MM, Sabatini DM, Yilmaz OH (2014). Dietary and metabolic control of stem cell function in physiology and cancer. Cell Stem Cell.

[86] Warburg O, Wind F, Negelein E (1927). The metabolism of tumors in the body. J Gen Physiol.

[87] Sun H, Chen L, Cao S, Liang Y, Xu Y (2019). Warburg effects in cancer and normal proliferating cells: two tales of the same name. Genom Proteom Bioinf.

[88] Basak O, van de Born M, Korving J, Beumer J, van der Elst S, van Es JH, Clevers H (2014). Mapping early fate determination in Lgr5+ crypt stem cells using a novel Ki67-RFP allele. EMBO J.

[89] McCabe LR, Parameswaran N (2017). Recent advances in intestinal stem cells. Curr Mol Biol Rep.

[90] Schell JC, Wisidagama DR, Bensard C, Zhao H, Wei P, Tanner J, Flores A, Mohlman J, Sorensen LK, Earl CS (2017). Control of intestinal stem cell function and proliferation by mitochondrial pyruvate metabolism. Nat Cell Biol.

[91] Fan YY, Davidson LA, Callaway ES, Wright GA, Safe S, Chapkin RS (2015). A bioassay to measure energy metabolism in mouse colonic crypts, organoids, and sorted stem cells. Am J Physiol Gastrointest Liver Physiol.

[92] Wei P, Dove KK, Bensard C, Schell JC, Rutter J (2018). The force is strong with this one: metabolism (Over)powers stem cell fate. Trends Cell Biol.

[93] Vander Heiden MG, DeBerardinis RJ (2017). Understanding the intersections between metabolism and cancer biology. Cell.

[94] Rodriguez-Colman MJ, Schewe M, Meerlo M, Stigter E, Gerrits J, Pras-Raves M, Sacchetti A, Hornsveld M, Oost KC, Snippert HJ (2017). Interplay between metabolic identities in the intestinal crypt supports stem cell function. Nature.

[95] Beyaz S, Mana MD, Roper J, Kedrin D, Saadatpour A, Hong SJ, Bauer-Rowe KE, Xifaras ME, Akkad A, Arias E (2016). High-fat diet enhances stemness and tumorigenicity of intestinal progenitors. Nature.

[96] Wang B, Rong X, Palladino END, Wang J, Fogelman AM, Martin MG, Alrefai WA, Ford DA, Tontonoz P (2018). Phospholipid remodeling and cholesterol availability regulate intestinal stemness and tumorigenesis. Cell Stem Cell.

[97] La Vecchia S, Sebastian C (2020). Metabolic pathways regulating colorectal cancer initiation and progression. Semin Cell Dev Biol.

[98] Pavlova NN, Thompson CB (2016). The emerging hallmarks of cancer metabolism. Cell Metab.

[99] Hutton JE, Wang X, Zimmerman LJ, Slebos RJ, Trenary IA, Young JD, Li M, Liebler DC (2016). Oncogenic KRAS and BRAF drive metabolic reprogramming in colorectal cancer. Mol Cell Proteomics.

[100] Munoz-Pinedo C, El Mjiyad N, Ricci JE (2012). Cancer metabolism: current perspectives and future directions. Cell Death Dis.

[101] Harris AL (2002). Hypoxia—a key regulatory factor in tumour growth. Nat Rev Cancer.

[102] Vaupel P, Mayer A (2007). Hypoxia in cancer: significance and impact on clinical outcome. Cancer Metastasis Rev.

[103] Berra E, Milanini J, Richard DE, Le Gall M, Vinals F, Gothie E, Roux D, Pages G, Pouyssegur J (2000). Signaling angiogenesis via p42/p44 MAP kinase and hypoxia. Biochem Pharmacol.

[104] Blancher C, Moore JW, Talks KL, Houlbrook S, Harris AL (2000). Relationship of hypoxia-inducible factor (HIF)-1alpha and HIF-2alpha expression to vascular endothelial growth factor induction and hypoxia survival in human breast cancer cell lines. Cancer Res.

[105] Mitsushita J, Lambeth JD, Kamata T (2004). The superoxide-generating oxidase Nox1 is functionally required for Ras oncogene transformation. Cancer Res.

[106] Mizukami Y, Fujiki K, Duerr EM, Gala M, Jo WS, Zhang X, Chung DC (2006). Hypoxic regulation of vascular endothelial growth factor through the induction of phosphatidylinositol 3-kinase/Rho/ROCK and c-Myc. J Biol Chem.

[107] Wang Y, Lei F, Rong W, Zeng Q, Sun W (2015). Positive feedback between oncogenic KRAS and HIF-1alpha confers drug resistance in colorectal cancer. Onco Targets Ther.

[108] Zeng M, Kikuchi H, Pino MS, Chung DC (2010). Hypoxia activates the K-ras proto-oncogene to stimulate angiogenesis and inhibit apoptosis in colon cancer cells. PLoS ONE.

[109] Kikuchi H, Pino MS, Zeng M, Shirasawa S, Chung DC (2009). Oncogenic KRAS and BRAF differentially regulate hypoxia-inducible factor-1alpha and -2alpha in colon cancer. Cancer Res.

[110] Chun SY, Johnson C, Washburn JG, Cruz-Correa MR, Dang DT, Dang LH (2010). Oncogenic KRAS modulates mitochondrial metabolism in human colon cancer cells by inducing HIF-1alpha and HIF-2alpha target genes. Mol Cancer.

[111] Hanahan D, Weinberg RA (2000). The hallmarks of cancer. Cell.

[112] Ramanathan A, Wang C, Schreiber SL (2005). Perturbational profiling of a cell-line model of tumorigenesis by using metabolic measurements. Proc Natl Acad Sci U S A.

[113] Brahimi-Horn MC, Chiche J, Pouyssegur J (2007). Hypoxia and cancer. J Mol Med (Berl).

[114] Semenza GL (2007). Hypoxia-inducible factor 1 (HIF-1) pathway. Sci STKE.

[115] Dang DT, Chen F, Gardner LB, Cummins JM, Rago C, Bunz F, Kantsevoy SV, Dang LH (2006). Hypoxia-inducible factor-1alpha promotes nonhypoxia-mediated proliferation in colon cancer cells and xenografts. Cancer Res.

[116] Commisso C, Davidson SM, Soydaner-Azeloglu RG, Parker SJ, Kamphorst JJ, Hackett S, Grabocka E, Nofal M, Drebin JA, Thompson CB (2013). Macropinocytosis of protein is an amino acid supply route in Ras-transformed cells. Nature.

[117] Recouvreux MV, Commisso C (2017). Macropinocytosis: a metabolic adaptation to nutrient stress in cancer. Front Endocrinol (Lausanne).

[118] Kamphorst JJ, Nofal M, Commisso C, Hackett SR, Lu W, Grabocka E, Vander Heiden MG, Miller G, Drebin JA, Bar-Sagi D (2015). Human pancreatic cancer tumors are nutrient poor and tumor cells actively scavenge extracellular protein. Cancer Res.

[119] Tejeda-Munoz N, Albrecht LV, Bui MH, De Robertis EM (2019). Wnt canonical pathway activates macropinocytosis and lysosomal degradation of extracellular proteins. Proc Natl Acad Sci U S A.

[120] Nusse R, Clevers H (2017). Wnt/beta-catenin signaling, disease, and emerging therapeutic modalities. Cell.

[121] Lushchak VI (2014). Free radicals, reactive oxygen species, oxidative stress and its classification. Chem Biol Interact.

[122] Janssen-Heininger YM, Mossman BT, Heintz NH, Forman HJ, Kalyanaraman B, Finkel T, Stamler JS, Rhee SG, van der Vliet A (2008). Redox-based regulation of signal transduction: principles, pitfalls, and promises. Free Radic Biol Med.

[123] Porporato PE, Payen VL, Perez-Escuredo J, De Saedeleer CJ, Danhier P, Copetti T, Dhup S, Tardy M, Vazeille T, Bouzin C (2014). A mitochondrial switch promotes tumor metastasis. Cell Rep.

[124] Acharya A, Das I, Chandhok D, Saha T (2010). Redox regulation in cancer: a double-edged sword with therapeutic potential. Oxid Med Cell Longev.

[125] Ziech D, Franco R, Pappa A, Panayiotidis MI (2011). Reactive oxygen species (ROS)–induced genetic and epigenetic alterations in human carcinogenesis. Mutat Res.

[126] Crespo-Sanjuan J, Calvo-Nieves MD, Aguirre-Gervas B, Herreros-Rodriguez J, Velayos-Jimenez B, Castro-Alija MJ, Munoz-Moreno MF, Sanchez D, Zamora-Gonzalez N, Bajo-Graneras R (2015). Early detection of high oxidative activity in patients with adenomatous intestinal polyps and colorectal adenocarcinoma: myeloperoxidase and oxidized low-density lipoprotein in serum as new markers of oxidative stress in colorectal cancer. Lab Med.

[127] Liu H, Liu X, Zhang C, Zhu H, Xu Q, Bu Y, Lei Y (2017). Redox imbalance in the development of colorectal cancer. J Cancer.

[128] Ogrunc M (2014). Reactive oxygen species: the good, the bad, and the enigma. Mol Cell Oncol.

[129] Lim JKM, Leprivier G (2019). The impact of oncogenic RAS on redox balance and implications for cancer development. Cell Death Dis.

[130] Wu RF, Terada LS (2009). Ras and Nox: linked signaling networks?. Free Radic Biol Med.

[131] Laurent E, McCoy JW, Macina RA, Liu W, Cheng G, Robine S, Papkoff J, Lambeth JD (2008). Nox1 is over-expressed in human colon cancers and correlates with activating mutations in K-Ras. Int J Cancer.

[132] Park MT, Kim MJ, Suh Y, Kim RK, Kim H, Lim EJ, Yoo KC, Lee GH, Kim YH, Hwang SG (2014). Novel signaling axis for ROS generation during K-Ras-induced cellular transformation. Cell Death Differ.

[133] Arber N, Eagle CJ, Spicak J, Racz I, Dite P, Hajer J, Zavoral M, Lechuga MJ, Gerletti P, Tang J (2006). Celecoxib for the prevention of colorectal adenomatous polyps. N Engl J Med.

[134] Oshima M, Dinchuk JE, Kargman SL, Oshima H, Hancock B, Kwong E, Trzaskos JM, Evans JF, Taketo MM (1996). Suppression of intestinal polyposis in Apc delta716 knockout mice by inhibition of cyclooxygenase 2 (COX-2). Cell.

[135] Jacoby RF, Cole CE, Tutsch K, Newton MA, Kelloff G, Hawk ET, Lubet RA (2000). Chemopreventive efficacy of combined piroxicam and difluoromethylornithine treatment of Apc mutant Min mouse adenomas, and selective toxicity against Apc mutant embryos. Cancer Res.

[136] Maciag A, Sithanandam G, Anderson LM (2004). Mutant K-rasV12 increases COX-2, peroxides and DNA damage in lung cells. Carcinogenesis.

[137] Dubois RN, Abramson SB, Crofford L, Gupta RA, Simon LS, Van De Putte LB, Lipsky PE (1998). Cyclooxygenase in biology and disease. FASEB J.

[138] Wang D, Dubois RN (2010). The role of COX-2 in intestinal inflammation and colorectal cancer. Oncogene.

[139] Sinicrope FA, Gill S (2004). Role of cyclooxygenase-2 in colorectal cancer. Cancer Metastasis Rev.

[140] Wang D, Buchanan FG, Wang H, Dey SK, DuBois RN (2005). Prostaglandin E2 enhances intestinal adenoma growth via activation of the Ras-mitogen-activated protein kinase cascade. Cancer Res.

[141] Kaidi A, Qualtrough D, Williams AC, Paraskeva C (2006). Direct transcriptional up-regulation of cyclooxygenase-2 by hypoxia-inducible factor (HIF)-1 promotes colorectal tumor cell survival and enhances HIF-1 transcriptional activity during hypoxia. Cancer Res.

[142] Devenport SN, Shah YM (2019). Functions and implications of autophagy in colon cancer. Cells.

[143] Avalos Y, Pena-Oyarzun D, Budini M, Morselli E, Criollo A (2017). New roles of the primary cilium in autophagy. Biomed Res Int.

[144] Burada F, Nicoli ER, Ciurea ME, Uscatu DC, Ioana M, Gheonea DI (2015). Autophagy in colorectal cancer: an important switch from physiology to pathology. World J Gastrointest Oncol.

[145] Guo JY, Chen HY, Mathew R, Fan J, Strohecker AM, Karsli-Uzunbas G, Kamphorst JJ, Chen G, Lemons JM, Karantza V (2011). Activated Ras requires autophagy to maintain oxidative metabolism and tumorigenesis. Genes Dev.

[146] Guo JY, Karsli-Uzunbas G, Mathew R, Aisner SC, Kamphorst JJ, Strohecker AM, Chen G, Price S, Lu W, Teng X (2013). Autophagy suppresses progression of K-ras-induced lung tumors to oncocytomas and maintains lipid homeostasis. Genes Dev.

[147] Yang ZJ, Chee CE, Huang S, Sinicrope FA (2011). The role of autophagy in cancer: therapeutic implications. Mol Cancer Ther.

[148] Mathew R, Khor S, Hackett SR, Rabinowitz JD, Perlman DH, White E (2014). Functional role of autophagy-mediated proteome remodeling in cell survival signaling and innate immunity. Mol Cell.

[149] Lock R, Roy S, Kenific CM, Su JS, Salas E, Ronen SM, Debnath J (2011). Autophagy facilitates glycolysis during Ras-mediated oncogenic transformation. Mol Biol Cell.

[150] Zhang L, Yu J (2013). Role of apoptosis in colon cancer biology, therapy, and prevention. Curr Colorectal Cancer Rep.

[151] Kasper S, Breitenbuecher F, Reis H, Brandau S, Worm K, Kohler J, Paul A, Trarbach T, Schmid KW, Schuler M (2013). Oncogenic RAS simultaneously protects against anti-EGFR antibody-dependent cellular cytotoxicity and EGFR signaling blockade. Oncogene.

[152] Mohammad RM, Muqbil I, Lowe L, Yedjou C, Hsu HY, Lin LT, Siegelin MD, Fimognari C, Kumar NB, Dou QP (2015). Broad targeting of resistance to apoptosis in cancer. Semin Cancer Biol.

[153] Arlt A, Bauer I, Schafmayer C, Tepel J, Muerkoster SS, Brosch M, Roder C, Kalthoff H, Hampe J, Moyer MP (2009). Increased proteasome subunit protein expression and proteasome activity in colon cancer relate to an enhanced activation of nuclear factor E2-related factor 2 (Nrf2). Oncogene.

[154] Almond JB, Cohen GM (2002). The proteasome: a novel target for cancer chemotherapy. Leukemia.

[155] Okamoto K, Zaanan A, Kawakami H, Huang S, Sinicrope FA (2015). Reversal of mutant KRAS-mediated apoptosis resistance by concurrent Noxa/Bik Induction and Bcl-2/Bcl-xL antagonism in colon cancer cells. Mol Cancer Res.

[156] Zaanan A, Okamoto K, Kawakami H, Khazaie K, Huang S, Sinicrope FA (2015). The mutant KRAS Gene Up-regulates BCL-XL protein via STAT3 to confer apoptosis resistance that is reversed by BIM protein induction and BCL-XL antagonism. J Biol Chem.

[157] Falschlehner C, Emmerich CH, Gerlach B, Walczak H (2007). TRAIL signalling: decisions between life and death. Int J Biochem Cell Biol.

[158] Ashkenazi A, Pai RC, Fong S, Leung S, Lawrence DA, Marsters SA, Blackie C, Chang L, McMurtrey AE, Hebert A (1999). Safety and antitumor activity of recombinant soluble Apo2 ligand. J Clin Invest.

[159] Walczak H, Miller RE, Ariail K, Gliniak B, Griffith TS, Kubin M, Chin W, Jones J, Woodward A, Le T (1999). Tumoricidal activity of tumor necrosis factor-related apoptosis-inducing ligand in vivo. Nat Med.

[160] von Karstedt S, Conti A, Nobis M, Montinaro A, Hartwig T, Lemke J, Legler K, Annewanter F, Campbell AD, Taraborrelli L (2015). Cancer cell-autonomous TRAIL-R signaling promotes KRAS-driven cancer progression, invasion, and metastasis. Cancer Cell.

[161] Strater J, Walczak H, Pukrop T, Von Muller L, Hasel C, Kornmann M, Mertens T, Moller P (2002). TRAIL and its receptors in the colonic epithelium: a putative role in the defense of viral infections. Gastroenterology.

[162] Hoogwater FJ, Nijkamp MW, Smakman N, Steller EJ, Emmink BL, Westendorp BF, Raats DA, Sprick MR, Schaefer U, Van Houdt WJ (2010). Oncogenic K-Ras turns death receptors into metastasis-promoting receptors in human and mouse colorectal cancer cells. Gastroenterology.

[163] von Karstedt S, Walczak H (2020). An unexpected turn of fortune: targeting TRAIL-Rs in KRAS-driven cancer. Cell Death Discov.

[164] Yahaya MAF, Lila MAM, Ismail S, Zainol M, Afizan N (2019). Tumour-associated macrophages (TAMs) in colon cancer and how to reeducate them. J Immunol Res.

[165] Dias Carvalho P, Guimaraes CF, Cardoso AP, Mendonca S, Costa AM, Oliveira MJ, Velho S (2018). KRAS oncogenic signaling extends beyond cancer cells to orchestrate the microenvironment. Cancer Res.

[166] Mueller L, Goumas FA, Affeldt M, Sandtner S, Gehling UM, Brilloff S, Walter J, Karnatz N, Lamszus K, Rogiers X (2007). Stromal fibroblasts in colorectal liver metastases originate from resident fibroblasts and generate an inflammatory microenvironment. Am J Pathol.

[167] Herrera M, Herrera A, Dominguez G, Silva J, Garcia V, Garcia JM, Gomez I, Soldevilla B, Munoz C, Provencio M (2013). Cancer-associated fibroblast and M2 macrophage markers together predict outcome in colorectal cancer patients. Cancer Sci.

[168] Kalluri R (2016). The biology and function of fibroblasts in cancer. Nat Rev Cancer.

[169] Monteran L, Erez N (1835). The dark side of fibroblasts: cancer-associated fibroblasts as mediators of immunosuppression in the tumor microenvironment. Front Immunol.

[170] Santos AM, Jung J, Aziz N, Kissil JL, Pure E (2009). Targeting fibroblast activation protein inhibits tumor stromagenesis and growth in mice. J Clin Invest.

[171] Henry LR, Lee HO, Lee JS, Klein-Szanto A, Watts P, Ross EA, Chen WT, Cheng JD (2007). Clinical implications of fibroblast activation protein in patients with colon cancer. Clin Cancer Res.

[172] Liu T, Han C, Wang S, Fang P, Ma Z, Xu L, Yin R (2019). Cancer-associated fibroblasts: an emerging target of anti-cancer immunotherapy. J Hematol Oncol.

[173] Peddareddigari VG, Wang D, Dubois RN (2010). The tumor microenvironment in colorectal carcinogenesis. Cancer Microenviron.

[174] De Boeck A, Hendrix A, Maynard D, Van Bockstal M, Daniels A, Pauwels P, Gespach C, Bracke M, De Wever O (2013). Differential secretome analysis of cancer-associated fibroblasts and bone marrow-derived precursors to identify microenvironmental regulators of colon cancer progression. Proteomics.

[175] Esfandi F, Mohammadzadeh Ghobadloo S, Basati G (2006). Interleukin-6 level in patients with colorectal cancer. Cancer Lett.

[176] Galizia G, Orditura M, Romano C, Lieto E, Castellano P, Pelosio L, Imperatore V, Catalano G, Pignatelli C, De Vita F (2002). Prognostic significance of circulating IL-10 and IL-6 serum levels in colon cancer patients undergoing surgery. Clin Immunol.

[177] Zeng J, Tang ZH, Liu S, Guo SS (2017). Clinicopathological significance of overexpression of interleukin-6 in colorectal cancer. World J Gastroenterol.

[178] Morris JP, Wang SC, Hebrok M (2010). KRAS, Hedgehog Wnt and the twisted developmental biology of pancreatic ductal adenocarcinoma. Nat Rev Cancer.

[179] Mills LD, Zhang Y, Marler RJ, Herreros-Villanueva M, Zhang L, Almada LL, Couch F, Wetmore C, Pasca di Magliano M, Fernandez-Zapico ME (2013). Loss of the transcription factor GLI1 identifies a signaling network in the tumor microenvironment mediating KRAS oncogene-induced transformation. J Biol Chem.

[180] Sternberg C, Gruber W, Eberl M, Tesanovic S, Stadler M, Elmer DP, Schlederer M, Grund S, Roos S, Wolff F (2018). Synergistic cross-talk of hedgehog and interleukin-6 signaling drives growth of basal cell carcinoma. Int J Cancer.

[181] Vainer N, Dehlendorff C, Johansen JS (2018). Systematic literature review of IL-6 as a biomarker or treatment target in patients with gastric, bile duct, pancreatic and colorectal cancer. Oncotarget.

[182] Lauth M, Bergstrom A, Shimokawa T, Toftgard R (2007). Inhibition of GLI-mediated transcription and tumor cell growth by small-molecule antagonists. Proc Natl Acad Sci U S A.

[183] Qualtrough D, Buda A, Gaffield W, Williams AC, Paraskeva C (2004). Hedgehog signalling in colorectal tumour cells: induction of apoptosis with cyclopamine treatment. Int J Cancer.

[184] Mazumdar T, DeVecchio J, Agyeman A, Shi T, Houghton JA (2011). The GLI genes as the molecular switch in disrupting Hedgehog signaling in colon cancer. Oncotarget.

[185] Yoshikawa K, Shimada M, Miyamoto H, Higashijima J, Miyatani T, Nishioka M, Kurita N, Iwata T, Uehara H (2009). Sonic hedgehog relates to colorectal carcinogenesis. J Gastroenterol.

[186] Douard R, Moutereau S, Pernet P, Chimingqi M, Allory Y, Manivet P, Conti M, Vaubourdolle M, Cugnenc PH, Loric S (2006). Sonic Hedgehog-dependent proliferation in a series of patients with colorectal cancer. Surgery.

[187] Bian YH, Huang SH, Yang L, Ma XL, Xie JW, Zhang HW (2007). Sonic hedgehog-Gli1 pathway in colorectal adenocarcinomas. World J Gastroenterol.

[188] Varnat F, Siegl-Cachedenier I, Malerba M, Gervaz P, Ruiz i Altaba A (2010). Loss of WNT-TCF addiction and enhancement of HH-GLI1 signalling define the metastatic transition of human colon carcinomas. EMBO Mol Med.

[189] Janssen KP, Alberici P, Fsihi H, Gaspar C, Breukel C, Franken P, Rosty C, Abal M, El Marjou F, Smits R (2006). APC and oncogenic KRAS are synergistic in enhancing Wnt signaling in intestinal tumor formation and progression. Gastroenterology.

[190] Sadot E, Geiger B, Oren M, Ben-Ze'ev A (2001). Down-regulation of beta-catenin by activated p53. Mol Cell Biol.

[191] Song L, Li ZY, Liu WP, Zhao MR (2015). Crosstalk between Wnt/beta-catenin and Hedgehog/Gli signaling pathways in colon cancer and implications for therapy. Cancer Biol Ther.

[192] Galon J, Costes A, Sanchez-Cabo F, Kirilovsky A, Mlecnik B, Lagorce-Pages C, Tosolini M, Camus M, Berger A, Wind P (2006). Type, density, and location of immune cells within human colorectal tumors predict clinical outcome. Science.

[193] Klampfer L, Huang J, Corner G, Mariadason J, Arango D, Sasazuki T, Shirasawa S, Augenlicht L (2003). Oncogenic Ki-ras inhibits the expression of interferon-responsive genes through inhibition of STAT1 and STAT2 expression. J Biol Chem.

[194] Klampfer L (2006). Signal transducers and activators of transcription (STATs): Novel targets of chemopreventive and chemotherapeutic drugs. Curr Cancer Drug Targets.

[195] Lal N, White BS, Goussous G, Pickles O, Mason MJ, Beggs AD, Taniere P, Willcox BE, Guinney J, Middleton GW (2018). KRAS Mutation and consensus molecular subtypes 2 and 3 are independently associated with reduced immune infiltration and reactivity in colorectal cancer. Clin Cancer Res.

[196] Khan S, Cameron S, Blaschke M, Moriconi F, Naz N, Amanzada A, Ramadori G, Malik IA (2014). Differential gene expression of chemokines in KRAS and BRAF mutated colorectal cell lines: role of cytokines. World J Gastroenterol.

[197] Huber S, Gagliani N, Zenewicz LA, Huber FJ, Bosurgi L, Hu B, Hedl M, Zhang W, O'Connor W, Murphy AJ (2012). IL-22BP is regulated by the inflammasome and modulates tumorigenesis in the intestine. Nature.

[198] Kirchberger S, Royston DJ, Boulard O, Thornton E, Franchini F, Szabady RL, Harrison O, Powrie F (2013). Innate lymphoid cells sustain colon cancer through production of interleukin-22 in a mouse model. J Exp Med.

[199] Wang C, Gong G, Sheh A, Muthupalani S, Bryant EM, Puglisi DA, Holcombe H, Conaway EA, Parry NAP, Bakthavatchalu V (2017). Interleukin-22 drives nitric oxide-dependent DNA damage and dysplasia in a murine model of colitis-associated cancer. Mucosal Immunol.

[200] Kryczek I, Lin Y, Nagarsheth N, Peng D, Zhao L, Zhao E, Vatan L, Szeliga W, Dou Y, Owens S (2014). IL-22(+)CD4(+) T cells promote colorectal cancer stemness via STAT3 transcription factor activation and induction of the methyltransferase DOT1L. Immunity.

[201] McCuaig S, Barras D, Mann EH, Friedrich M, Bullers SJ, Janney A, Garner LC, Domingo E, Koelzer VH, Delorenzi M (2020). The interleukin 22 pathway interacts with mutant KRAS to promote poor prognosis in colon cancer. Clin Cancer Res.

[202] Schroder K, Hertzog PJ, Ravasi T, Hume DA (2004). Interferon-gamma: an overview of signals, mechanisms and functions. J Leukoc Biol.

[203] Petanidis S, Anestakis D, Argyraki M, Hadzopoulou-Cladaras M, Salifoglou A (2013). Differential expression of IL-17, 22 and 23 in the progression of colorectal cancer in patients with K-ras mutation: Ras signal inhibition and crosstalk with GM-CSF and IFN-gamma. PLoS ONE.

[204] Hanggi K, Ruffell B (2019). Oncogenic KRAS drives immune suppression in colorectal cancer. Cancer Cell.

[205] Chang SH, Mirabolfathinejad SG, Katta H, Cumpian AM, Gong L, Caetano MS, Moghaddam SJ, Dong C (2014). T helper 17 cells play a critical pathogenic role in lung cancer. Proc Natl Acad Sci U S A.

[206] Grivennikov SI, Wang K, Mucida D, Stewart CA, Schnabl B, Jauch D, Taniguchi K, Yu GY, Osterreicher CH, Hung KE (2012). Adenoma-linked barrier defects and microbial products drive IL-23/IL-17-mediated tumour growth. Nature.

[207] Neurath MF (2019). IL-23 in inflammatory bowel diseases and colon cancer. Cytokine Growth Factor Rev.

[208] Sparmann A, Bar-Sagi D (2004). Ras-induced interleukin-8 expression plays a critical role in tumor growth and angiogenesis. Cancer Cell.

[209] Vidal M, Cusick ME, Barabasi AL (2011). Interactome networks and human disease. Cell.

[210] Kolch W, Halasz M, Granovskaya M, Kholodenko BN (2015). The dynamic control of signal transduction networks in cancer cells. Nat Rev Cancer.

[211] Huttlin EL, Bruckner RJ, Paulo JA, Cannon JR, Ting L, Baltier K, Colby G, Gebreab F, Gygi MP, Parzen H (2017). Architecture of the human interactome defines protein communities and disease networks. Nature.

[212] Kennedy SA, Jarboui MA, Srihari S, Raso C, Bryan K, Dernayka L, Charitou T, Bernal-Llinares M, Herrera-Montavez C, Krstic A (2020). Extensive rewiring of the EGFR network in colorectal cancer cells expressing transforming levels of KRAS(G13D). Nat Commun.

[213] Global Burden of Disease Cancer C, Fitzmaurice C, Allen C, Barber RM, Barregard L, Bhutta ZA, Brenner H, Dicker DJ, Chimed-Orchir O, Dandona R et al. Global, Regional, and National Cancer Incidence, Mortality, Years of Life Lost, Years Lived With Disability, and Disability-Adjusted Life-years for 32 Cancer Groups, 1990 to 2015: A Systematic Analysis for the Global Burden of Disease Study. JAMA Oncol 2017, 3(4):524–48. 10.1001/jamaoncol.2016.5688 PMC6103527 27918777

[214] Canon J, Rex K, Saiki AY, Mohr C, Cooke K, Bagal D, Gaida K, Holt T, Knutson CG, Koppada N (2019). The clinical KRAS(G12C) inhibitor AMG 510 drives anti-tumour immunity. Nature.

[215] Tran NH, Cavalcante LL, Lubner SJ, Mulkerin DL, LoConte NK, Clipson L, Matkowskyj KA, Deming DA (2015). Precision medicine in colorectal cancer: the molecular profile alters treatment strategies. Ther Adv Med Oncol.

[216] Binefa G, Rodriguez-Moranta F, Teule A, Medina-Hayas M (2014). Colorectal cancer: from prevention to personalized medicine. World J Gastroenterol.

[217] Altunel E, Roghani RS, Chen KY, Kim SY, McCall S, Ware KE, Shen X, Somarelli JA, Hsu DS (2020). Development of a precision medicine pipeline to identify personalized treatments for colorectal cancer. BMC Cancer.

[218] Prasad B, Vrana M, Mehrotra A, Johnson K, Bhatt DK (2017). The promises of quantitative proteomics in precision medicine. J Pharm Sci.

[219] Barbolosi D, Ciccolini J, Lacarelle B, Barlesi F, Andre N (2016). Computational oncology–mathematical modelling of drug regimens for precision medicine. Nat Rev Clin Oncol.

[220] Bernard C (1965). An introduction to the study of experimental medicine. Med J Aust.

